# Isoliquiritigenin Attenuates Adipose Tissue Inflammation *in vitro* and Adipose Tissue Fibrosis through Inhibition of Innate Immune Responses in Mice

**DOI:** 10.1038/srep23097

**Published:** 2016-03-15

**Authors:** Yasuharu Watanabe, Yoshinori Nagai, Hiroe Honda, Naoki Okamoto, Seiji Yamamoto, Takeru Hamashima, Yoko Ishii, Miyako Tanaka, Takayoshi Suganami, Masakiyo Sasahara, Kensuke Miyake, Kiyoshi Takatsu

**Affiliations:** 1 Department of Immunobiology and Pharmacological Genetics, Graduate School of Medicine and Pharmaceutical Science for Research, University of Toyama, 2630 Sugitani, Toyama-shi, Toyama 930-0194, JAPAN; 2 JST, PRESTO, 4-1-8 Honcho, Kawaguchi, Saitama 332-0012, JAPAN; 3 Toyama Prefectural Institute for Pharmaceutical Research, 17-1 Nakataikouyama, Imizu City, Toyama 939-0363, JAPAN; 4 Department of Pathology, Graduate School of Medicine and Pharmaceutical Science for Research, University of Toyama, 2630 Sugitani, Toyama-shi, Toyama 930-0194, JAPAN; 5 Department of Molecular Medicine and Metabolism, Research Institute of Environmental Medicine, Nagoya University, Furo-cho, Chikusa-ku, Nagoya 464-8601, JAPAN; 6 Division of Infectious Genetics, Department of Microbiology and Immunology, The Institute of Medical Science, The University of Tokyo, 4-6-1 Shirokanedai, Minato-ku, Tokyo 108-8639, JAPAN; 7 Laboratory of Innate Immunity, Center for Experimental Medicine and Systems Biology, The Institute of Medical Science, The University of Tokyo, 4-6-1 Shirokanedai, Minato-ku, Tokyo 108-8639, JAPAN

## Abstract

Isoliquiritigenin (ILG) is a flavonoid derived from *Glycyrrhiza uralensis* and potently suppresses NLRP3 inflammasome activation resulting in the improvement of diet-induced adipose tissue inflammation. However, whether ILG affects other pathways besides the inflammasome in adipose tissue inflammation is unknown. We here show that ILG suppresses adipose tissue inflammation by affecting the paracrine loop containing saturated fatty acids and TNF-α by using a co-culture composed of adipocytes and macrophages. ILG suppressed inflammatory changes induced by the co-culture through inhibition of NF-κB activation. This effect was independent of either inhibition of inflammasome activation or activation of peroxisome proliferator-activated receptor-γ. Moreover, ILG suppressed TNF-α-induced activation of adipocytes, coincident with inhibition of IκBα phosphorylation. Additionally, TNF-α-mediated inhibition of Akt phosphorylation under insulin signaling was alleviated by ILG in adipocytes. ILG suppressed palmitic acid-induced activation of macrophages, with decreasing the level of phosphorylated Jnk expression. Intriguingly, ILG improved high fat diet-induced fibrosis in adipose tissue *in vivo*. Finally, ILG inhibited TLR4- or Mincle-stimulated expression of fibrosis-related genes in stromal vascular fraction from obese adipose tissue and macrophages *in vitro*. Thus, ILG can suppress adipose tissue inflammation by both inflammasome-dependent and -independent manners and attenuate adipose tissue fibrosis by targeting innate immune sensors.

Accumulating evidence suggests that obesity is associated with a state of chronic low-grade inflammation with infiltration of macrophages into adipose tissue, leading to insulin resistance^1^,^2^. Adipose tissue inflammation is increased by adipocyte lipolysis and adipose tissue fibrosis in obesity^3^. One study used a co-culture of adipocytes and macrophages to demonstrate a paracrine loop composed of saturated fatty acids (FAs) and TNF-α derived from adipocytes and macrophages, respectively^4^. Adipocyte derived endogenous FAs activate macrophages and induce TNF-α production, which in turn stimulates adipocytes and induces monocyte chemotactic protein-1 (MCP-1) expression in adipocytes, which is crucial for macrophage infiltration into adipose tissue^5^. Nuclear factor kappa light chain enhancer of activated B cells (NF-κB) and mitogen-activating protein (MAP) kinases, such as Erk (extracellular-signal-regulated kinase) and Jnk (c-Jun N-terminal kinase), are activated in the co-culture and play important roles in the induction of inflammatory changes in adipocytes and macrophages^4^,^6^.

The extracellular matrix is particularly important for maintaining the structural integrity of adipocytes and plays a critical role in adipogenesis. Fibrosis of adipose tissue may play an important role in adipose tissue dysfunction^3^. The connective fiber content of adipose tissue dramatically increases by the upregulation of collagen expression, which in turn elevates the overall rigidity of adipose tissue and finally leads to fibrosis. The deficiency of collagen 6, a key component of the extracellular matrix in adipose tissue, significantly improves the phenotypes of obese mice, including adipocyte death and adipose tissue inflammation^3^. This implies that alterations in the extracellular matrix in adipose tissue are linked to the development of inflammation. Thus, not only inflammation but also fibrosis may be important targets for the treatment of obesity.

Pattern recognition receptors (PRRs) such as Toll-like receptors (TLRs) quickly recognize pathogenic agents^7^. The TLR4/myeloid differentiation protein (MD)-2 complex is essential for lipopolysaccharide (LPS) recognition^8^,^9^. MD-2 reacts with LPS directly and this reaction induces homodimerization of the TLR4/MD-2 complex^10^. PRRs also recognize endogenous ligands called danger-associated molecular patterns (DAMPs). In the obese state, TLR4/MD-2 may recognize saturated free FAs, such as palmitic acid, derived from hypertrophied adipocytes as a DAMP and promote adipose tissue inflammation and insulin resistance^4^,^6^,^11^. Inflammasomes are multimeric protein complexes that are crucial for caspase-1 and IL-1β production^12^. The nucleotide-binding domain, leucine-rich repeats containing family, pyrin domain-containing-3 (NLRP3) inflammasome senses obesity-associated FAs and contributes to obesity-induced inflammation and insulin resistance^13^,^14^. Moreover, IL-1β inhibits insulin signaling in the insulin-target organs, including adipose tissue, liver, and skeletal muscle, and also induces dysfunction and cell death of insulin-producing pancreatic β cells^15^. Macrophage inducible C-type lectin (Mincle) recognizes not only cord factor, a mycobacterial glycolipid, but also SAP130 released from dead cells^16^,^17^. Furthermore, Mincle is highly expressed in M1 macrophages in adipose tissue and involved in the induction of adipose tissue fibrosis and insulin resistance during obesity^18^,^19^.

Isoliquiritigenin (ILG) is a component of *Glycyrrhiza uralensis* (*G. uralensis*) and a flavonoid with a chalcone structure. Its biological activities include anti-allergic^20^, anti-angiogenesis^21^, and anti-tumor growth^22^. A recent paper showed that ILG promotes regulatory T cell differentiation^23^. We reported previously that ILG suppressed LPS-TLR4/MD-2 complex signaling at the receptor level, resulting in inhibition of NF-κB and MAP kinases activation^24^. Furthermore, we found that ILG potently inhibited the activation of the NLRP3 inflammasome^25^. Our *in vivo* study revealed that the NLRP3 inflammasome of white adipose tissue (WAT) was an important target of ILG for improving diet-induced adipose tissue inflammation and insulin resistance^25^. However, it remains unclear whether ILG affects other pathways besides the inflammasome in adipose tissue inflammation. Additionally, little is known regarding whether ILG impacts fibrogenesis in adipose tissue.

This study explored the effects of ILG on adipose tissue inflammation and fibrosis. We now report that ILG suppressed adipose tissue inflammation by affecting the paracrine loop of the co-culture composed of adipocytes and macrophages. Furthermore, ILG markedly improved high fat diet (HFD)-induced adipose tissue fibrosis *in vivo*. We will discuss how ILG attenuates the inflammatory paracrine loop and adipose tissue fibrosis through inhibition of innate immune responses.

## Results

### Inflammatory responses in the co-culture of adipocytes and macrophages were suppressed by ILG stimulation independent of PPARγ activity

**Figure 1:**
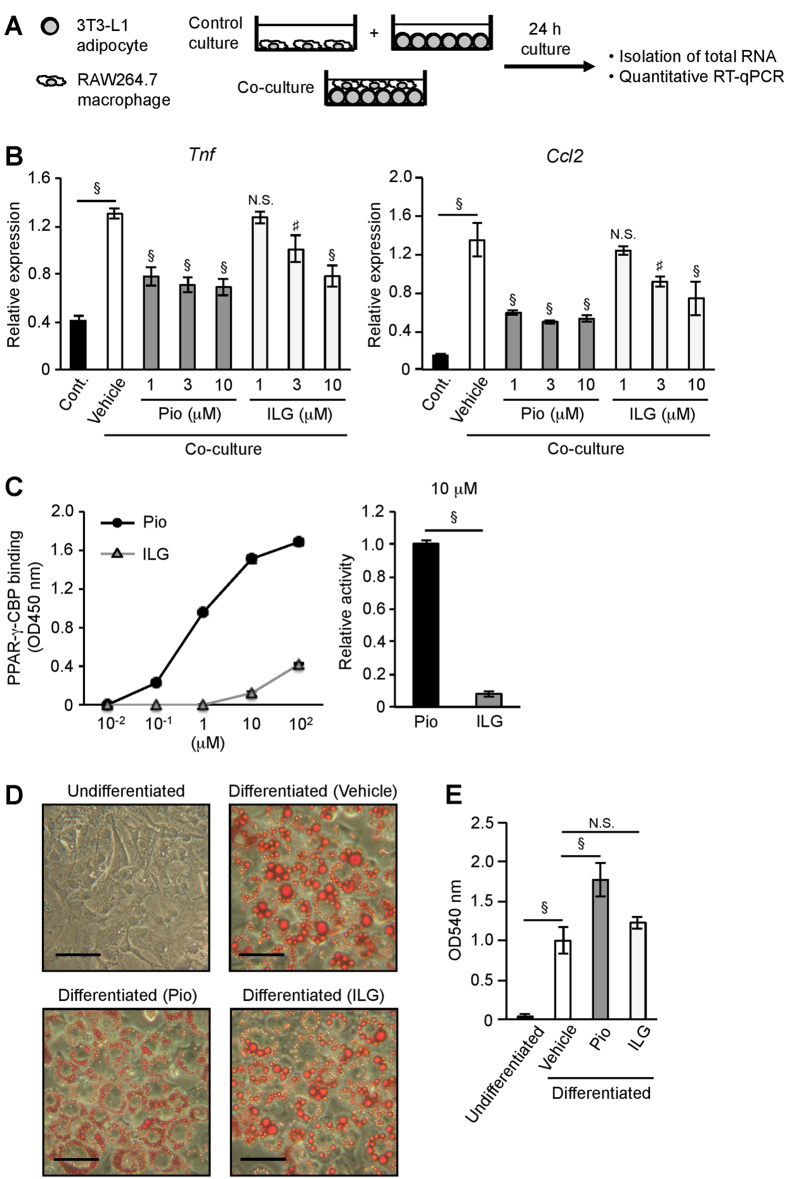
ILG suppresses inflammatory changes induced by the co-culture of adipocytes and macrophages. (**A**) Schematic diagram of the co-culture system. Differentiated 3T3-L1 adipocytes and RAW264.7 macrophages were co-cultured for 24 h. As a control culture, these cells were separately cultured and harvested for RT-qPCR. (**B**) RT-qPCR for TNF-α and MCP-1 mRNA in the control and co-cultured cells with or without pioglitazone (Pio) or ILG (n = 3 per group). Data are shown as means ± SD. N.S., not significant. ^#^*P* < 0.01, ^§^*P* < 0.001. (**C**) The interaction between CBP and PPARγ in the presence of pioglitazone (Pio) or ILG was determined as described in Methods. Data are shown as the mean ± SD. ^§^*P* < 0.001. (**D**) 3T3-L1 cells were differentiated in the presence of pioglitazone (Pio) or ILG for 8 days as described in Methods. Representative Oil-Red-O staining images of 3T3-L1 cells are shown (n = 3 per group). The scale bars are 50 μM. (**E**) Spectrophotometric analysis of Oil-Red-O staining. Data are shown as means ± SD. N.S., not significant. ^§^*P* < 0.001. All data are representative of at least three independent experiments.

To examine the effects of ILG on adipose tissue inflammation, we used a co-culture system composed of differentiated 3T3-L1 adipocytes and RAW264.7 macrophages^4^,^26^ (Fig. 1A). Compared with bone marrow-derived macrophages (BMMs), RAW264.7 had low levels of IL-1β mRNA expression and mature IL-1β production by LPS and LPS plus ATP stimulation, respectively (Supplementary Fig. 1A,B). Whereas IL-1β mRNA expression was marginally but not significantly increased in the co-cultured cells compared with cells in the control (Supplementary Fig. 1C), mature IL-1β was not significantly produced by the co-culture of differentiated 3T3-L1 and either RAW264.7 or BMMs (Supplementary Fig. 1D).

We observed increased expression of TNF-α and MCP-1 mRNA in the co-cultured cells compared with the control culture (Fig. 1B). These expressions were decreased by ILG stimulation, albeit at high concentrations. As we previously reported^26^, a PPARγ agonist pioglitazone also decreased TNF-α and MCP-1 mRNA expression induced by the co-culture. We then investigated PPARγ agonistic activity of ILG by measuring the interaction between CREB binding protein (CBP) and PPARγ. ILG displayed much lower binding of PPARγ to CBP than pioglitazone, and the binding activity of 10 μM ILG was only one tenth that of 10 μM pioglitazone (Fig. 1C). To verify the low PPARγ agonistic activity of ILG, we examined the effect of ILG on adipogenesis. However, ILG had little effect on adipogenesis of differentiated 3T3-L1 cells (Fig. 1D,E). In parallel with the high PPARγ agonistic activity, pioglitazone significantly increased adipogenesis. Although several studies have shown that ILG inhibits cell growth and induces apoptosis^27^,^28^,^29^, the cell viabilities of RAW264.7 cells and 3T3-L1 adipocytes were not affected by ILG at the concentrations used (Supplementary Fig. 2). Taking these findings together, ILG suppresses inflammasome-independent inflammation in the co-culture of adipocytes and macrophages *in vitro* and PPARγ agonistic activity is not involved in the inhibitory effects of ILG.

### ILG suppresses inflammatory changes elicited by the interaction between adipocytes and macrophages

**Figure 2:**
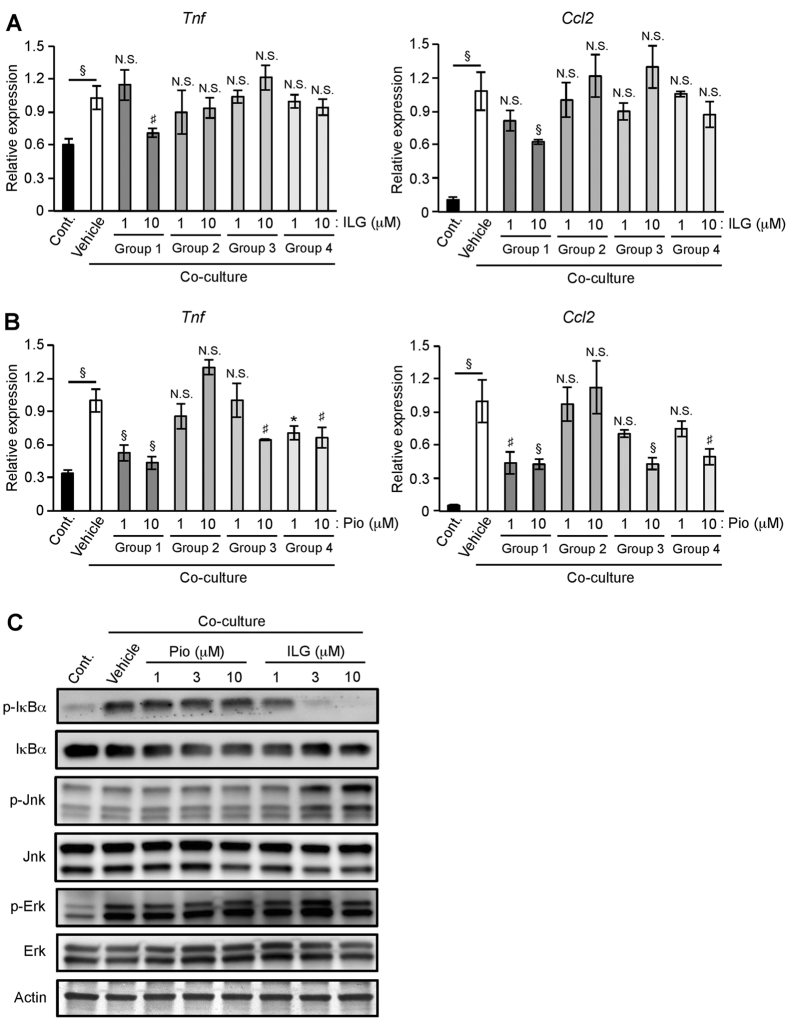
ILG suppresses inflammatory changes elicited by the interaction between adipocytes and macrophages and attenuates NF-κB activation induced by the co-culture. (**A**) Schematic diagram of the co-culture of differentiated 3T3-L1 adipocytes and RAW264.7 macrophages. (**A,B**) RT-qPCR for TNF-α and MCP-1 mRNA in the control and co-cultured cells with or without ILG (**A**) or pioglitazone (Pio) (**B**) (n = 3 per group). Data are shown as means ± SD. N.S., not significant. ^*^P < 0.05, ^#^*P* < 0.01, ^§^*P* < 0.001. All data are representative of at least three independent experiments. (**C**) Differentiated 3T3-L1 adipocytes and RAW264.7 macrophages were co-cultured in the presence of pioglitazone (Pio) or ILG for 1 h. Phosphorylation (p) of IκBα, Jnk, and Erk was examined by western blotting. All data are representative of at least three independent experiments.

To explore the mechanisms behind the inhibitory effects of ILG on the co-culture system, it was differently added to the culture of differentiated 3T3-L1 adipocytes and RAW264.7 macrophages as indicated in Supplementary Fig. 3. ILG stimulation decreased the levels of TNF-α and MCP-1 mRNA expression when it was added at the start of co-culture as shown in Fig. 1B (Fig. 2A, Group 1). In contrast, expressions of TNF-α and MCP-1 mRNA were not suppressed when ILG was added to either differentiated 3T3-L1 or RAW264.7 (Fig. 2A, Group 2 and Group 3). Those expressions were not affected when differentiated 3T3-L1 and RAW264.7 were treated with ILG individually before the co-culture (Fig. 2A, Group 4). These results indicate that ILG suppresses inflammatory changes elicited by the interaction between adipocytes and macrophages. Pioglitazone stimulation suppressed co-culture-induced TNF-α and MCP-1 mRNA expression when it was added at the start of co-culture as shown in Fig. 1B (Fig. 2B, Group 1). However, when RAW264.7 was treated with pioglitazone for 24 h before the co-culture, those expressions were not significantly affected (Fig. 2B, Group 2). In contrast, expressions of TNF-α and MCP-1 mRNA were decreased when pioglitazone was added to differentiated 3T3-L1 only or both differentiated 3T3-L1 and RAW264.7 before the co-culture (Fig. 2B, Group 3 and Group 4).

### ILG attenuates NF-κB activation induced by the co-culture of adipocytes and macrophages

We then investigated the molecular mechanisms by which ILG suppressed inflammation in the co-culture. As NF-κB plays a critical role in the co-culture-induced inflammatory changes^6^, we firstly examined whether ILG affected the co-culture-induced NF-κB activation. We observed the phosphorylation of IκBα in the co-cultured cells compared with the control cells (Fig. 2C and Supplementary Fig. 4). This phosphorylation was potently inhibited by 3 and 10 μM of ILG. ILG stimulation also inhibited the co-culture-induced IκBα degradation (Fig. 2C). In contrast, the phosphorylation of IκBα was not obviously affected by pioglitazone stimulation (Fig. 2C and Supplementary Fig. 4). Activation of MAP kinases is also important for the induction of inflammatory changes of adipocytes and macrophages^4^. Phosphorylation of Erk was seen in the co-cultured cells compared with the control cells, whereas neither ILG nor pioglitazone stimulation inhibited this phosphorylation. Jnk and p38 MAP kinases were not phosphorylated by the co-culture compared with the control culture (Fig. 2C and data not shown).

### ILG blunts TNF-α-induced insulin resistance in adipocytes through inhibition of NF-κB activation

**Figure 3:**
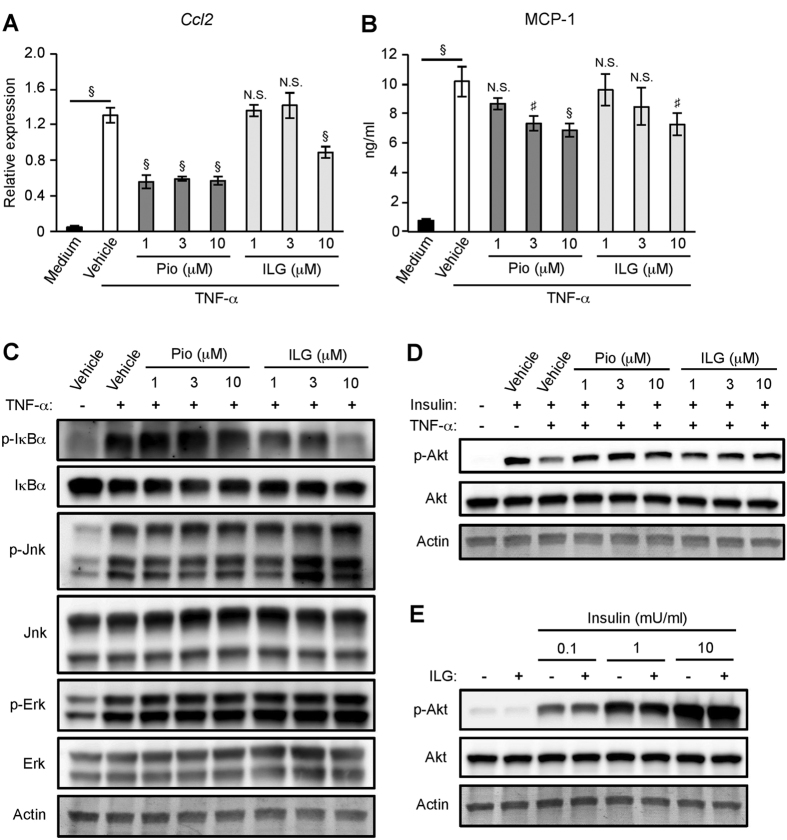
ILG restores TNF-α-induced inflammatory changes and inhibition of insulin signaling in adipocytes. (**A,B**) Differentiated 3T3-L1 adipocytes were treated with pioglitazone (Pio) or ILG for 6 h and subsequently stimulated with TNF-α (5 ng/ml) for 24 h (n = 3 per group). MCP-1 mRNA expression (**A**) and secretion (**B**) were measured by RT-qPCR and ELISA, respectively. Data are shown as means ± SD. N.S., not significant. ^#^*P* < 0.01, ^§^*P* < 0.001 (Medium vs. Vehicle, Vehicle vs. Pioglitazone or ILG). (**C**) Differentiated 3T3-L1 adipocytes were treated with pioglitazone (Pio) or ILG for 6 h and subsequently stimulated with TNF-α (1 ng/ml) for 10 min. Phosphorylation (p) of IκBα, Jnk, and Erk was examined by western blotting. (**D**) Differentiated 3T3-L1 adipocytes were treated with pioglitazone (Pio) or ILG for 6 h and subsequently stimulated with TNF-α (5 ng/ml) for 18 h. The cultured cells were treated with insulin for 30 min before ending of culture. Phosphorylation (p) of Akt was examined by western blotting. (**E**) Differentiated 3T3-L1 adipocytes were treated with ILG (10 μM) for 6 h and subsequently treated with indicated concentration of insulin for 30 min before ending of culture. Phosphorylation (p) of Akt was examined by western blotting. All data are representative of at least three independent experiments.

TNF-α secreted by macrophages plays a critical role in the induction of inflammatory changes in adipocytes^4^. Stimulation of differentiated 3T3-L1 adipocytes with TNF-α induced MCP-1 mRNA expression and secretion (Fig. 3A,B). A high concentration of ILG (10 μM) significantly suppressed TNF-α-induced MCP-1 mRNA expression and secretion. Pioglitazone also potently decreased TNF-α-induced MCP-1 mRNA expression and secretion. Activation of NF-κB and MAP kinases plays critical roles in TNF-α-induced pro-inflammatory changes in adipocytes^4^,^6^. Phosphorylation and degradation of IκBα were observed in TNF-α-stimulated 3T3-L1 adipocytes (Fig. 3C and Supplementary Fig. 5A). ILG potently inhibited TNF-α-induced IκBα phosphorylation relative to pioglitazone. It was reported that NF-κB activation induced by TNF-α was not affected by pioglitazone stimulation^30^. Consistent with this report, TNF-α-induced IκBα phosphorylation was poorly inhibited by 10 μM of pioglitazone stimulation. Phosphorylation of Jnk and Erk was also observed in TNF-α-stimulated 3T3-L1 adipocytes (Fig. 3C). However, neither ILG nor pioglitazone had inhibitory effects on these changes.

TNF-α stimulation induces insulin resistance through activation of NF-κB and MAP kinases in adipocytes^31^. Then, we examined whether ILG inhibits TNF-α-induced insulin resistance in adipocytes. TNF-α stimulation decreased insulin-induced Akt phosphorylation in differentiated 3T3-L1 adipocytes (Fig. 3D). ILG was effective in attenuating the reduction of Akt phosphorylation (Fig. 3D and Supplementary Fig. 5B). Although pioglitazone stimulation poorly affects TNF-α-induced NF-κB activation in adipocytes (Fig. 3C), Akt phosphorylation was restored by the stimulation (Fig. 3D and Supplementary Fig. 5B). We further examined whether ILG directly enhanced insulin signaling. Insulin-induced Akt phosphorylation was not augmented by even a high concentration of ILG (10 μM) (Fig. 3E and Supplementary Fig. 5C). Thus, ILG alleviates TNF-α-mediated insulin resistance in adipocytes, presumably through inhibition of NF-κB activation.

### ILG but not pioglitazone suppresses palmitic acid-induced macrophage activation

**Figure 4:**
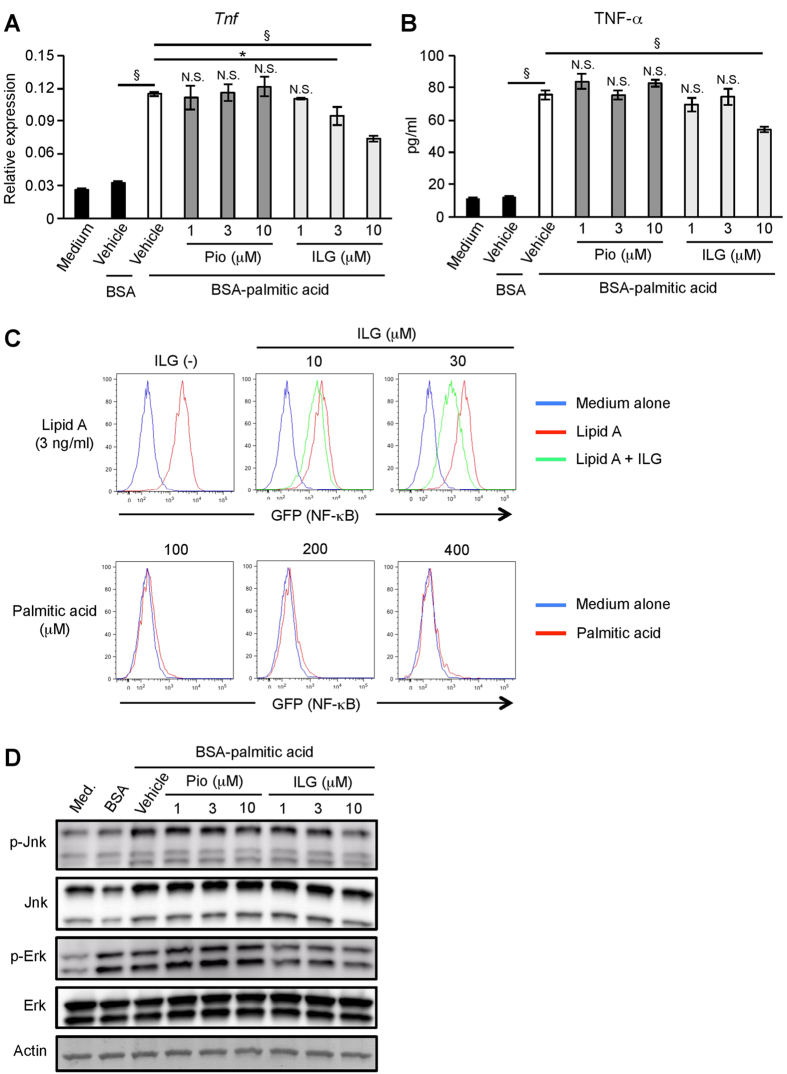
ILG suppresses palmitic acid-induced TLR4 activation in macrophages. (**A,B**) BMMs were treated with pioglitazone (Pio) or ILG for 6 h and subsequently stimulated with BSA-palmitic acid (200 μM) for 24 h (n = 3 per group). TNF-α mRNA expression (**A**) and secretion (**B**) were measured by RT-qPCR and ELISA, respectively. Data are shown as means ± SD. N.S., not significant. ^*^P < 0.05, ^§^*P* < 0.001. (**C**, upper panel) Ba/F3 cells expressing murine TLR4/MD-2 and CD14 were treated with ILG (10 or 30 μM) for 1 h and subsequently stimulated with lipid A (3 ng/ml) for 18 h. The cells were harvested and GFP expression was monitored by flow cytometry. (Lower panel) Ba/F3 cells expressing murine TLR4/MD-2 and CD14 were stimulated with the indicated concentrations of BSA-palmitic acid for 18 h. The cells were harvested and GFP expression was monitored by flow cytometry. (**D**) BMMs were treated with pioglitazone (Pio) or ILG for 6 h and subsequently stimulated with BSA-palmitic acid (200 μM) for 2 h (n = 3 per group). Phosphorylation (p) of Jnk and Erk was examined by western blotting. All data are representative of at least three independent experiments.

Saturated FAs such as palmitic acid are important adipocyte-derived mediators of macrophage activation in co-culture^4^. Palmitic acid-induced macrophage activation was dependent on TLR4 signaling^26^. Furthermore, palmitic acid activates the NF-κB pathway through TLR4^6^. Additionally, ILG attenuates lipid A, a biologically active component of LPS, -induced NF-κB activation^24^. Therefore, we explored the effects of ILG on palmitic acid-induced TLR4 activation. We firstly examined whether ILG affected FA release from adipocytes. TNF-α stimulation induced FA release from differentiated 3T3-L1 adipocytes in a dose-dependent manner (Supplementary Fig. 6). Whereas pioglitazone significantly decreased the FA release, ILG had no effect on this response. This suggests that PPARγ agonistic activity may be important for the inhibition of FA release from adipocytes. Then, we examined the effects of ILG on palmitic acid stimulation by using BMMs. BSA-conjugated palmitic acid (BSA-palmitic acid), but not BSA alone, significantly increased TNF-α mRNA and secretion in BMMs as previously reported (Fig.4A,B)^26^. ILG decreased BSA-palmitic acid-induced TNF-α mRNA expression in a dose-dependent manner (Fig. 4A). TNF-α secretion induced by BSA-palmitic acid was also significantly suppressed by 10 μM of ILG (Fig. 4B). In contrast, pioglitazone had no effect on these responses.

Since the TLR4/NF-κB pathway plays a critical role in the saturated FA-induced inflammatory responses^6^, we examined the phosphorylation and degradation of IκBα in BSA-palmitic acid-stimulated BMMs by western blotting. However, IκBα phosphorylation and degradation in BMMs were not induced by even a high concentration of BSA-palmitic acid (100~400 μM) (data not shown). So we examined NF-κB activation by using Ba/F3 cells expressing murine TLR4/MD-2 complex and CD14^24^. The Ba/F3 cells were pretreated with ILG and then stimulated with lipid A or palmitic acid (Fig. 4C). NF-κB activation in the cultured cells was monitored by measuring GFP expression from a reporter construct using flow cytometry. Lipid A stimulation induced NF-κB activation and ILG pre-treatment inhibited its activation in a dose-dependent manner, as we previously reported (Fig. 4C, upper panel)^24^. In contrast, we did not observe NF-κB activation by BSA-palmitic acid stimulation (Fig. 4C, lower panel).

MAP kinases also play an important role in palmitic acid-induced TNF-α expression^4^. Phosphorylation of Jnk was observed in BSA-palmitic acid-stimulated BMMs (Fig. 4D). This phosphorylation was inhibited by ILG but not pioglitazone stimulation (Fig. 4D and Supplementary Fig. 7A). BSA-palmitic acid stimulation appeared to phosphorylate Erk, but its phosphorylation was also observed at BSA alone (Fig. 4D and Supplementary Fig. 7B). Phosphorylated p38 MAP kinase was not observed by BSA-palmitic acid stimulation (data not shown). These results indicate that ILG suppresses palmitic acid-induced TNF-α expression and production, presumably through inhibition of Jnk phosphorylation.

### ILG attenuates LPS plus IFN-γ-induced differentiation to M1 macrophages

*In vivo* treatment of ILG to HFD-fed mice reduced the numbers of M1 and M2 macrophages in epididymal white adipose tissue (eWAT)^25^. Therefore, we examined the effects of ILG on the differentiation of M1 and M2 macrophages. 10 μM of ILG strongly suppressed the expression of M1 markers, including iNOS and TNF-α, in LPS plus IFN-γ-stimulated BMMs (Supplementary Fig. 8A). Pioglitazone treatment of HFD-fed mice decreased M1 markers and increased M2 markers in eWAT^32^. Consistent with this, pioglitazone suppressed the expression of M1 markers (Supplementary Fig. 8A). ILG had no effect on the expression of the M2 markers including arginase 1 and CD206 in IL-4-stimulated BMMs (Supplementary Fig. 8B). In contrast, pioglitazone significantly increased the expression of the M2 markers.

### ILG supplementation improves HFD-induced adipose tissue fibrosis with decreased expression of TLR4 and Mincle

**Figure 5:**
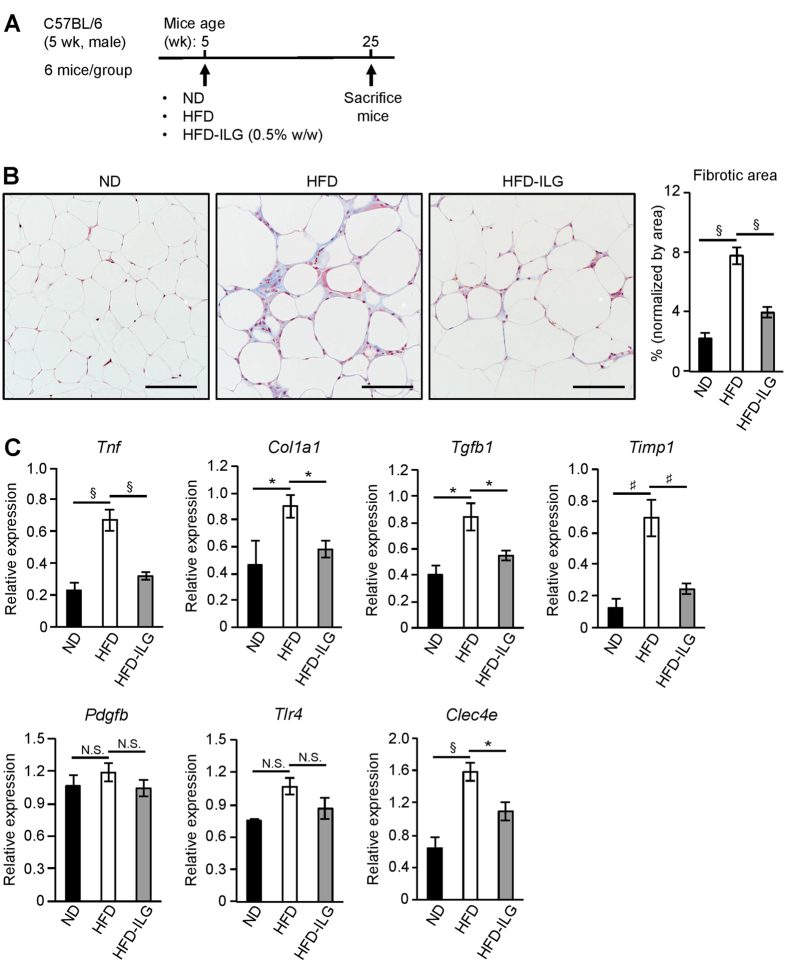
ILG supplementation attenuates HFD-induced adipose tissue fibrosis and expression of fibrosis-related genes in eWAT. (**A**) Schematic diagram of the experiment of ILG treatment to HFD-fed mice. (**B**) Representative images of Masson trichrome-stained sections of eWAT from mice fed with ND, HFD, or HFD-ILG for 20 weeks. Scale bars, 100 μm. Fibrotic area was analyzed by KEYENCE BZ-II Analyzer software. (**C**) RT-qPCR of TNF-α, collagen type 1, TGF-β, TIMP-1, PDGF-B, TLR4, and Mincle mRNA in eWAT from WT mice fed with ND, HFD or HFD-ILG for 20 weeks (n = 3 per group). Data are shown as the mean ± SE. N.S., not significant. ^*^P < 0.05, ^#^*P* < 0.01, ^§^*P* < 0.001. Data are representative of at least two independent experiments.

Adipose tissue exhibits interstitial fibrosis under the state of chronic inflammation or during the development of obesity^3^. Since ILG supplementation markedly improves HFD-induced adipose tissue inflammation and insulin resistance^25^, we examined whether ILG improves HFD-induced adipose tissue fibrosis. Male 5-week old C57BL/6 mice were fed a HFD, HFD supplemented with ILG (0.5% w/w; HFD-ILG) or normal diet (ND) for 20 weeks (Fig. 5A). Histological analysis clearly demonstrated that HFD induced extensive interstitial fibrosis in eWAT, which was markedly suppressed by ILG supplementation (Fig. 5B). HFD increased TNF-α and collagen 1 mRNA expression in eWAT (Fig. 5C). These expressions were markedly decreased by ILG supplementation, suggesting that ILG inhibits HFD-induced adipose tissue inflammation and fibrosis. HFD also increased TGF-β and TIMP-1 mRNA expression, which regulate extracellular matrix (ECM) production and ECM degradation, respectively. ILG supplementation markedly suppressed the increases of these expressions. Expression of PDGF-B, which regulates fibroblast proliferation, and TLR4 mRNA were marginally but not significantly increased by HFD. These increases were also marginally suppressed by ILG supplementation. Macrophage-inducible C-type lectin (Mincle) is an innate immune sensor that recognizes fungi and *Mycobacterium tuberculosis*^16^,^33^,^34^,^35^. Mincle is expressed selectively in adipose tissue macrophages^18^ and its expression was markedly up-regulated during obesity^19^. We confirmed that Mincle (*Clec4e*) mRNA expression in eWAT was up-regulated by HFD. Furthermore, ILG supplementation significantly decreased HFD-induced Mincle mRNA expression in eWAT. Additionally, the co-culture of differentiated 3T3-L1 adipocytes and RAW264.7 macrophages increased the levels of TLR4 and Mincle mRNA expression, which were suppressed by ILG stimulation in a dose-dependent manner (Supplementary Fig. 9).

### ILG decreases TLR4- or Mincle-stimulated expression of fibrosis-related genes in SVF

**Figure 6:**
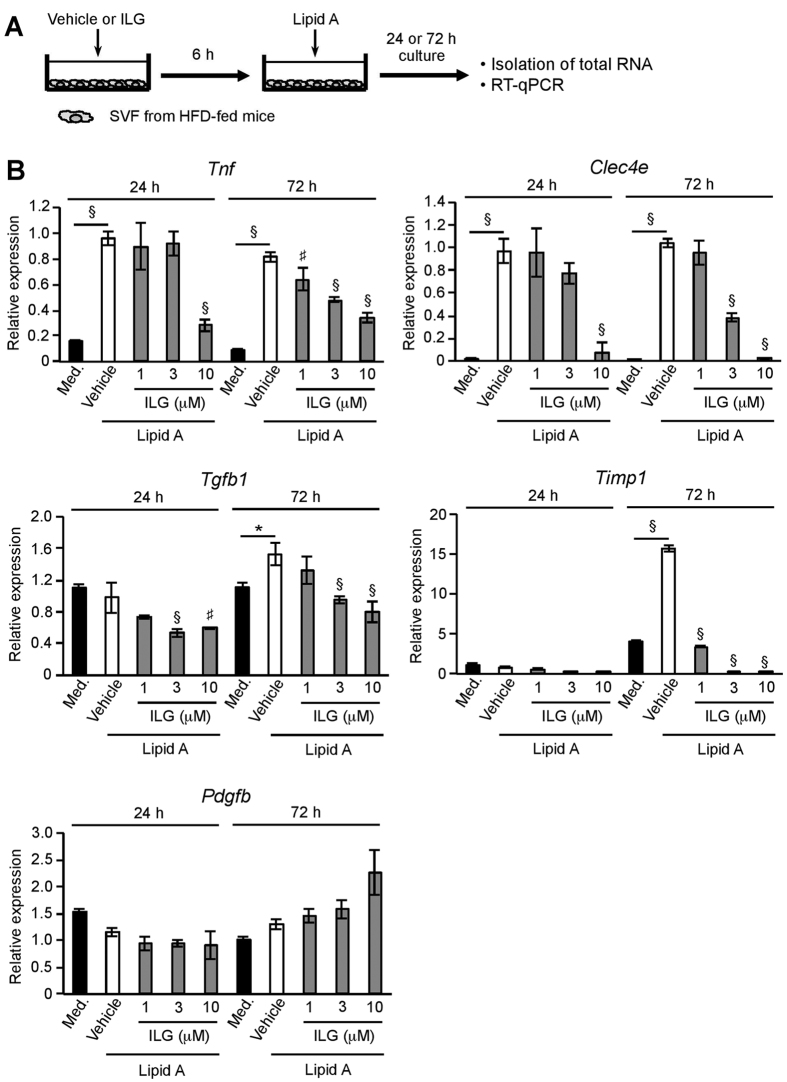
ILG attenuates TLR4-stimulated expression of fibrosis-related genes in SVF. (**A**) Schematic diagram of the stimulation of SVF. SVF from C57BL/6 mice fed with an HFD for 12 weeks was treated with vehicle or ILG for 6 h and subsequently stimulated with lipid A for 24 or 72 h. (**B**) RT-qPCR for TNF-α, Mincle, TGF-β, TIMP-1, and PDGF-B mRNA in the lipid A-stimulated SVF with or without ILG (n = 3 per group). Data are shown as the mean ± SE. ^*^P < 0.05, ^#^*P* < 0.01, ^§^*P* < 0.001. Data are representative of at least two independent experiments.

TLR4 signaling in immune cells plays a key role in the development of obesity- and endotoxin-mediated adipose tissue fibrosis^36^. We examined whether ILG attenuated TLR4-stimulated expression of fibrosis-related genes in stromal vascular fraction (SVF) of obese eWAT (Fig. 6A). Lipid A stimulation for 24 h significantly increased TNF-α and Mincle mRNA expression in the SVF (Fig. 6B). Lipid A stimulation also increased the expression of TNF-α, Mincle, TGF-β, and TIMP-1 mRNA at 72 h stimulation. These increases were significantly attenuated by ILG stimulation. Expression of PDGF-B mRNA was not affected by lipid A stimulation or ILG stimulation.

Mincle stimulation is also crucial for fibrogenesis in SVF of obese adipose tissue^18^. The SVF from HFD-fed mice was stimulated with a Mincle ligand trehalose-6,6′-dimycolate (TDM), a mycobacterial cell wall glycolipid (Supplementary Fig. 10A). TDM stimulation for 24 h significantly increased TNF-α, Mincle, TGF-β, and PDGF-B mRNA expression in the SVF (Supplementary Fig. 10B). The effect of TDM was also observed at 72 h stimulation in the expression of TNF-α, Mincle, and TIMP-1. These increases were significantly attenuated by ILG stimulation. It was reported that mRNA expression of *Acta2*, which encodes the myofibroblast marker αSMA, was increased by TDM stimulation^18^. However, we did not confirm this increase of *Acta2* (data not shown).

### ILG decreases TLR4- or Mincle-stimulated fibrosis-related gene expression in macrophages, presumably through inhibition of NF-κB pathway

**Figure 7:**
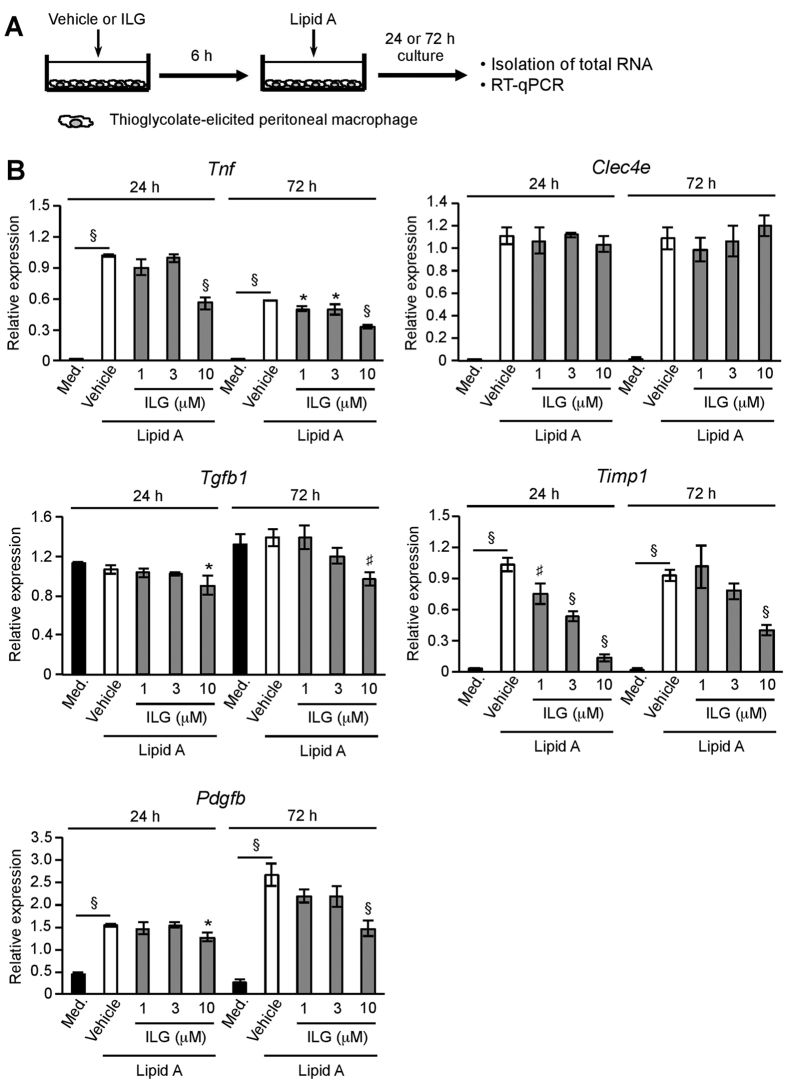
ILG attenuates TLR4-stimulated expression of fibrosis-related genes in macrophages. (**A**) Schematic diagram of the stimulation of macrophages. Thioglycolate-elicited peritoneal macrophages from C57BL/6 mice were treated with vehicle or ILG for 6 h and subsequently stimulated with lipid A for 24 or 72 h. (**B**) RT-qPCR for TNF-α, Mincle, TGF-β, TIMP-1, and PDGF-B mRNA in the lipid A-stimulated macrophages with or without ILG (n = 3 per group). Data are shown as the mean ± SE. ^*^P < 0.05, ^#^*P* < 0.01, ^§^*P* < 0.001. Data are representative of at least two independent experiments.

Finally, we explored the mechanisms behind the inhibitory effects of ILG on fibrogenesis. Activation of TLR4 and Mincle in monocytes or macrophages is crucial for the induction of fibrosis-related gene expression^18^,^37^. We examined whether ILG attenuated TLR4-stimulated their expression in macrophages (Fig. 7A). Lipid A stimulation for 24 or 72 h markedly increased TNF-α, TIMP-1, and PDGF-B mRNA expression in thioglycolate-elicited peritoneal macrophages (Fig. 7B). These increased expressions were significantly attenuated by ILG stimulation. Additionally, palmitic acid stimulation markedly increased Mincle mRNA expression in macrophages, which was significantly suppressed by ILG but not pioglitazone stimulation (Supplementary Fig. 11). Meanwhile, ILG had no effect on Mincle expression in lipid A-stimulated macrophages (Fig. 7B). Expression of TGF-β mRNA in macrophages was not increased by lipid A stimulation. A NF-κB inhibitor BAY11-7082 significantly suppressed lipid A-induced TNF-α and TIMP-1 mRNA expression at 24 and 72 h stimulation (Supplementary Fig. 12). In contrast, a MAP kinase inhibitor PD98059 exhibited weak suppression of TNF-α and TIMP-1 mRNA expression compared with BAY11-7082. ILG (30 μM) suppresses lipid A-induced phosphorylation of IκBα and MAP kinases in macrophages^24^. Western blotting analysis revealed that a lower concentration of ILG (3~10 μM) suppressed lipid A-induced phosphorylation of IκBα, Jnk, and p38 but not Erk (Supplementary Fig. 13 and Supplementary Fig. 14), which is effective in decreasing TNF-α and fibrosis-related gene expression (Fig. 7B).

We further examined whether ILG attenuates Mincle-stimulated fibrosis-related gene expression in macrophages (Supplementary Fig. 15A). TDM stimulation for 24 h significantly increased TNF-α, Mincle, TIMP-1, and PDGF-B mRNA expression in thioglycolate-elicited peritoneal macrophages (Supplementary Fig. 15B). The effect of TDM was also observed at 72 h stimulation in the expression of TNF-α, Mincle, and TIMP-1 mRNA. These increases were significantly attenuated by ILG stimulation in a dose-dependent manner. Similar to lipid A stimulation, TGF-β mRNA expression was not increased by TDM stimulation. Additionally, BAY11-7082 potently suppressed TDM-induced TNF-α and TIMP-1 mRNA expression at 24 h stimulation relative to PD98059 (Supplementary Fig. 16).

## Discussion

**Figure 8:**
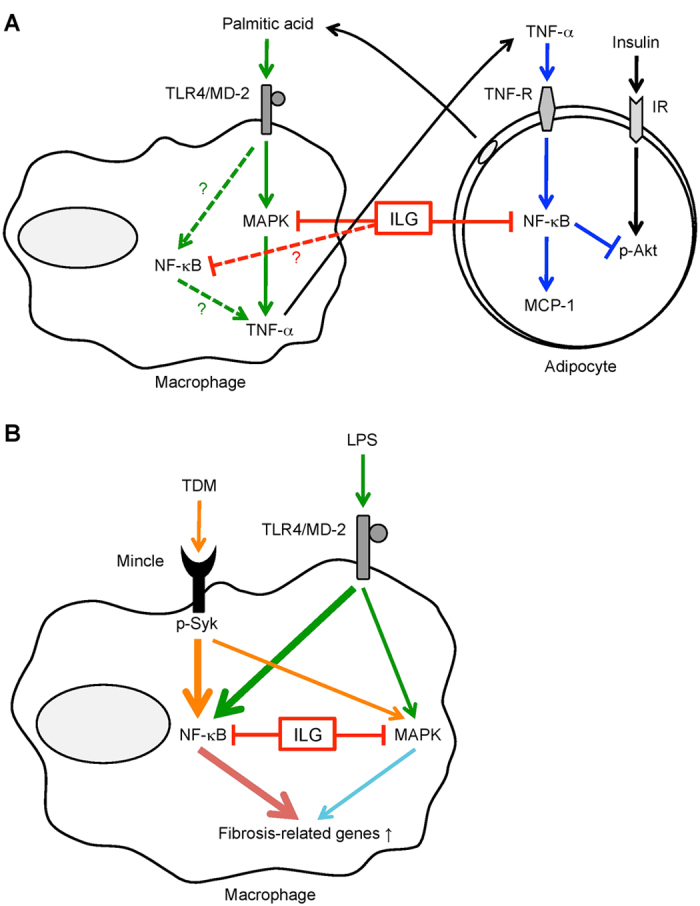
Proposed models of molecular targets of ILG in attenuating inflammation induced by the co-culture of adipocytes and macrophages and TLR4- or Mincle-stimulated expression of fibrosis-related genes in macrophages. (**A**) ILG suppresses TNF-α-induced MCP-1 mRNA expression and secretion in adipocytes, coincident with inhibition of NF-κB activation. In addition, ILG restores inhibition of Akt phosphorylation under insulin receptor signaling in TNF-α-stimulated adipocytes. ILG also suppresses palmitic acid-induced TNF-α mRNA and secretion in macrophages, with decreasing the level of phosphorylated MAPK (Jnk) expression. It remains unclear whether NF-κB is involved in palmitic acid-induced TNF-α mRNA and secretion in macrophages. (**B**) Lipid A- or TDM-induced expression of fibrosis-related genes such as TIMP-1 is potently suppressed by a NF-κB inhibitor BAY11-7082 relative to a MAP kinase inhibitor PD98059, suggesting that NF-κB may play a key role in the induction of those expressions. ILG inhibits TLR4- or Mincle-stimulated expression of fibrosis-related genes in macrophages, presumably through inhibition of NF-κB pathway.

We previously identified ILG as a potent inhibitor of NLRP3 inflammasome^25^. ILG supplementation inhibits IL-1β and caspase-1 production in obese eWAT^25^. Since ILG has various biological activities, we investigated whether ILG inhibits adipose tissue inflammation in an inflammasome-independent manner. We used a co-culture system composed of adipocytes and macrophages, which induces inflammatory responses via an inflammasome-independent fashion. The ILG blocked the inflammatory paracrine loop containing TNF-α and saturated FA derived from macrophages and adipocytes, respectively, and consequently inhibited inflammatory responses induced by the co-culture via a PPARγ-independent fashion. (Fig. 8A). In adipocytes, ILG inhibited activation of the NF-κB pathway induced by TNF-α. As a result, ILG attenuated TNF-α-stimulated insulin resistance in adipocytes. Additionally, ILG inhibited palmitic acid-induced TNF-α expression and production in macrophages, with inhibition of MAP kinase activation. In contrast, pioglitazone did not have such inhibitory effects on macrophages. ILG improved diet-induced fibrosis in obese eWAT, and ILG inhibited the TLR4- and Mincle-stimulated fibrogenic program in SVF of obese eWAT and peritoneal macrophages (Fig. 8B). Our new findings revealed that ILG could attenuate adipose tissue inflammation in an inflammasome-independent fashion by targeting the interaction between adipocytes and macrophages. ILG can also attenuate adipose tissue fibrosis by inhibiting activation of innate immune sensors.

ILG acts on adipocytes and macrophages, and consequently suppresses inflammatory changes elicited by the co-culture (Fig. 2A). Furthermore, ILG inhibits TNF-α- and palmitic acid-induced activation of adipocytes and macrophages, respectively (Figs 3 and 4). Thus, ILG has inhibitory effects on two major cell types, which are involved in the induction of adipose tissue inflammation. In contrast, pioglitazone predominantly acts on adipocytes, thereby suppressing inflammatory responses elicited by the co-culture (Fig. 2B). Although PPARγ is highly expressed in activated macrophages and has an anti-inflammatory function^38^,^39^, pioglitazone did not suppress palmitic acid-induced macrophages activation (Fig.4A,B).

Murine 3T3-L1 preadipocytes are a valuable cell line and have been widely used to study adipocyte biology. However, they are immortalized cells and have distinctive attributes compared with primary human preadipocytes. For instance, 3T3-L1 cells become resistant to apoptosis induced by growth factor deprivation during their differentiation^40^,^41^. In contrast, human primary preadipocytes become more sensitive to apoptotic stimuli as they differentiate into mature adipocytes *in vitro*^42^. Therefore, results based on murine adipocyte cell lines may not be applicable for human adipocyte study. An *in vitro* system may have some limitations to examine the effects of ILG on human adipocytes.

NF-κB plays an important role in the induction of MCP-1 expression and TNF-α-induced insulin resistance in adipocytes^6^,^43^. PPAR agonists inhibit inflammatory responses at multiple steps of the NF-κB pathway in both PPAR-dependent and PPAR-independent manners^44^,^45^. PPARγ agonistic activity of ILG was much lower than that of pioglitazone (Fig. 1C–E), suggesting that ILG may inhibit NF-κB pathway in a PPARγ-independent manner. ILG interacts with IKK directly and inhibits its kinase activity in TNF-α-stimulated endothelial cells^46^. Therefore, IKK is a potential target for the inhibitory effect of ILG on TNF-α-stimulated adipocytes.

Our new findings are in line with the previous observation that ILG inhibits ligand-induced activation of TLR4/MD-2 in macrophages^24^. ILG inhibits activation of TLR4/MD-2 at the receptor level, namely LPS-induced its homodimerization^24^. However, homodimerization of TLR4/MD-2 did not occur by palmitic acid stimulation^47^. This suggests that inhibitory effects of ILG on palmitic acid stimulation may not be attributed to inhibition of TLR4/MD-2 homodimerization. As ILG inhibits palmitic acid-induced Jnk phosphorylation (Fig. 4D) or interacts with IKK directly^46^, it is possible that ILG is incorporated intracellularly and blocks TLR4 signaling at multiple steps. Future studies will determine a molecular target of ILG action on palmitic acid-induced TLR4 activation.

NF-κB inhibitors inhibit palmitic acid-induced TNF-α mRNA expression and secretion in macrophages^6^, suggesting that activation of NF-κB may play a critical role in FA-induced TLR4 activation. However, we and others have demonstrated that NF-κB activation was not induced by palmitic acid stimulation in the Ba/F3 cells stably expressing TLR4/MD-2 and CD14 and BMMs (Fig. 4C and data not shown)^47^. Although palmitic acid has been suggested to bind to MD-2, ligand-dependent homodimerization was not induced by palmitic acid stimulation^47^. These data indicate that palmitic acid may induce weak activation of NF-κB via TLR4/MD-2. Further investigation will reveal the impact of ILG on the TLR4-NF-κB pathway in macrophages.

Excessive collagen accumulation and the development of fibrosis can limit adipose tissue expandability, resulting in increased and ectopic lipid accumulation and the development of metabolic syndrome^3^. Here we demonstrated for the first time that ILG attenuates diet-induced adipose tissue fibrosis. The NLRP3 inflammasome activation of eWAT is an important target of ILG for improving adipose tissue inflammation^25^. Therefore, ILG has multiple targets in obese adipose tissue for preventing the development of metabolic syndrome. Moreover, ILG may prevent the development of adipose tissue fibrosis at an early stage of HFD feeding, consequently improving NLRP3 inflammasome-stimulated adipose tissue inflammation.

PRRs such as TLRs and C-type lectin receptors elicit inflammation and innate immune responses through activation of multiple signaling cascades. Mincle transduces activation signals by associating with the Fc receptor common γ-chain (FcRγ), which contains immunoreceptor tyrosine-based activation motifs (ITAMs) in the cytoplasmic domain. The phosphorylated ITAM recruits spleen tyrosine kinase (Syk), leading to activation of NF-κB and MAP kinases^48^,^49^. Given that an inhibitor against NF-κB or Syk suppresses TDM-stimulated TIMP-1 expression (Supplementary Fig. 16)^18^, the Syk-NF-κB pathway may be crucial for Mincle-stimulated fibrogenesis. Additional analysis will determine the precise mechanisms by which ILG inhibits the activation of Mincle signaling.

The expression level of Mincle is increased by various cellular stresses and stimuli. Mincle expression was upregulated in patients with rheumatoid arthritis^50^,^51^. A recent paper demonstrated that Mincle expression was increased in microglia, neuron, and endothelial cells in the brain after ischemic stroke^51^. Furthermore, Syk was phosphorylated in ischemic brain and a Syk inhibitor successfully prevented ischemic brain injury^51^. These results suggest that Mincle may be involved in the pathogenesis of ischemic stroke by initiating inflammatory responses. Therefore, Mincle may represent a potential target molecule for the treatment of inflammatory diseases, including rheumatoid arthritis, brain infarction, type 2 diabetes. Intriguingly, ILG decreased Mincle expression induced by various treatments including HFD, lipid A, palmitic acid, TDM and the co-culture (Figs 5C,6B and Supplementary Figs 9,10,11,15). Thus, ILG may be useful for treating various inflammatory diseases.

In conclusion, we here show that ILG suppresses an inflammatory paracrine loop between adipocytes and macrophages in an inflammasome-independent fashion and attenuates adipose tissue fibrosis through inhibition of a variety of innate immune responses. Additionally, besides macrophages, adipocytes are key cells for the inhibitory effects of ILG on adipose tissue inflammation and insulin resistance. Our results suggest that ILG could be a candidate for a therapeutic agent to treat both adipose tissue inflammation and fibrosis in obesity.

## Methods

### Mice

C57BL/6 mice were purchased from Japan SLC (Hamamatsu, Japan) and were used at 8 to 10 weeks of age. C57BL/6 mice were maintained in microisolator cages under specific pathogen-free conditions, and maintained in the animal facility of University of Toyama. Male 5-week old C57BL/6 mice were fed a HFD containing 60% fat (Research Diet, New Brunswick, NJ, USA), HFD supplemented with ILG (0.5% w/w) or ND containing 10% fat (Research Diet) for 20 weeks^25^. The experimental protocols were approved by the Animal Studies Committees at University of Toyama and Toyama Prefectural Institute for Pharmaceutical Research, and all experiments were performed according to the guidelines for the care and treatment of experimental animals at the institutes.

### Reagents

ILG was purchased from AK Scientific Inc. (Union City, CA, USA) and Sigma-Aldrich (St. Louis, MO, USA). Pioglitazone was purchased from Funakoshi (Tokyo, Japan). ILG and pioglitazone were dissolved in dimethyl sulfoxide (DMSO). Recombinant mouse TNF-α, IL-4, and IFN-γ were purchased from R & D Systems (Minneapolis, MN, USA). Human insulin was purchased from Eli Lilly (Indianapolis, IN, USA). LPS from *E. coli* O55:B5, lipid A from *Salmonella minnesota*, palmitic acid, TDM, ATP, and thioglycolate were purchased from Sigma-Aldrich. Palmitic acid was conjugated to bovine serum albumin (BSA, Sigma-Aldrich) to increase solubility. BAY11-7082 was purchased from InvivoGen (San Diego, CA, USA). PD98059 was purchased from Merck Millipore (Darmstadt, Germany).

### Cell culture

RAW264.7 cells (RIKEN BioResource Center, Tsukuba, Japan) and 3T3-L1 cells (kindly provided by Drs. Takayoshi Suganami and Yoshihiro Ogawa, Tokyo Medical and Dental University) were maintained with DMEM containing 10% FCS and antibiotics, and incubated at 37˚C in a humidified 5% CO_2_. 3T3-L1 cells were differentiated based on a standard protocol^52^. Accumulated lipid droplets were visualized using Oil-Red-O staining (Cosmo-Bio, Tokyo, Japan). For spectrophotometric analysis, Oil-Red-O was eluted with 100% isopropanol and measured at 540 nm with a microplate reader (Bio-Rad, Hercules, CA, USA). BMMs and BMMs-derived M1 and M2 macrophages were prepared as described previously^26^. Ba/F3 cells expressing murine TLR4/MD-2 and CD14 were kindly provided by Dr. Kensuke Miyake (The University of Tokyo).

### PPARγ agonist activity

To measure PPARγ agonist activities of pioglitazone and ILG, the interaction between CBP and PPARγ was examined by using an ELISA kit (Fujikura Kasei, Tokyo, Japan) following the manufacture’s instructions.

### Non-esterified fatty acid (NEFA) measurements

NEFA in the culture supernatants were measured with a colorimetric assay (Wako, Osaka, Japan) according to the manufacturer’s instruction.

### Co-culture of adipocytes and macrophages

Co-culture of differentiated 3T3-L1 adipocytes and RAW264.7 macrophages was performed as described previously^6^. RAW264.7 macrophages were co-cultured with differentiated 3T3-L1 adipocytes in the absence or presence of pioglitazone or ILG for 24 h.

### TDM stimulation

Thioglycolate-elicited peritoneal macrophages were obtained from C57BL/6 mice that had been injected intraperitoneally 4 days before with 3% thioglycolate solution. Isolation of SVF from eWAT of HFD-fed C57/BL6 mice was performed as described previously^32^. TDM dissolved in chloroform at 1 mg/ml were diluted in isopropanol and added on 12-well plate (2.5 μg of TDM/well), followed by evaporation of the solvent as described^18^. SVF (2.5×10^5^/well) or peritoneal macrophages was cultured on the TDM coating plate in the absence or presence of ILG for 24 and 72 h.

### ELISA

Culture supernatants were collected, and levels of MCP-1, TNF-α, and IL-1β in the culture supernatants were determined by using ELISA kits (R & D Systems).

### Preparation of RNA and cDNA

Total RNA was isolated with RNeasy mini kit (QIAGEN, Hilden, Germany) and Sepasol® -RNA I Super G (Nacalai tesque, Kyoto, Japan) following the manufacture’s instructions. RNA was reverse transcribed with a TaqMan Reverse Transcription Reagents (Applied Biosystems, Carlsbad, CA, USA) following the manufacture’s instructions.

### Real-time quantitative PCR

RT-qPCR was performed with a TaqMan Gene Expression Master Mix (Applied Biosystems) and analyzed with a CFX96 Touch^TM^ Real-Time PCR Detection System (Bio-Rad) following the manufacture’s instructions. Relative transcript abundance was normalized for that of Hprt mRNA. The information for primers used for real-time PCR is listed in Supplementary Table 1.

### Western blotting analysis

Cells were washed and lysed for 60 min in iced lysis buffer containing 50 mM Tris-HCl pH 7.5, 150 mM NaCl, 1% Triton X-100, protease inhibitor cocktail (Nacalai tesque). Lysates were subjected to SDS-PAGE and western blotting analyses. The following antibodies for immunoblotting were purchased from Cell Signaling (Beverly, MA, USA): anti-IκBα, anti-phospho-IκB, anti-phospho-Jnk, anti-Jnk, anti-phospho-Erk, anti-Erk, anti-phospho-p38, anti-p38, anti-phospho-Akt, anti-Akt. Anti-actin was purchased from Sigma-Aldrich. Goat anti-rabbit IgG alkaline phosphatase was purchased from Bio-Rad. Goat anti-mouse IgG alkaline phosphatase was purchased from American Qualex (San Clemonte, CA, USA). The reactive bands were visualized by ECL Plus (GE Healthcare, Uppsala, Sweden). Relative protein levels were quantified by using the ImageJ.

### Cell viability assay

Cell viability assay was conducted by using a Cell Titer 96® Aqueous One Solution Cell Proliferation Assay (Promega, Madison, WI, USA).

### Histological analysis

Portions of the eWAT (n-2~3 per each group) were excised and fixed immediately with 4% formaldehyde at room temperature. Paraffin-embedded tissue sections were cut into 3-μm slices and placed on slides. Sections were stained with Masson trichrome stain. Twelve of eighteen images per each section were examined and fibrotic area was analyzed by KEYENCE BZ-II Analyzer software (KEYENCE, Osaka, Japan).

### Statistical analysis

Statistical significance was evaluated by one-way ANOVA followed by post-hoc Tukey test.

## Additional Information

**How to cite this article**: Watanabe, Y. *et al.* Isoliquiritigenin Attenuates Adipose Tissue Inflammation *in vitro* and Adipose Tissue Fibrosis through Inhibition of Innate Immune Responses in Mice. *Sci. Rep.* **6**, 23097; doi: 10.1038/srep23097 (2016).

## Supplementary Material

Supplementary Information
