# Supplementary material for: Isoliquiritigenin Attenuates Adipose Tissue Inflammation in vitro and Adipose Tissue Fibrosis through Inhibition of Innate Immune Responses in Mice
Source: Sci Rep. 2016 Mar 15;6:23097. doi: 10.1038/srep23097 (PMC4791553; doi:10.1038/srep23097)
Supplement: Supplementary Information [file srep23097-s1.pdf]

Supplementary Information

Isoliquiritigenin Attenuates Adipose Tissue inflammation *in vitro* and Adipose Tissue Fibrosis through Inhibition of Innate Immune Responses in Mice

Yasuharu Watanabe[1], Yoshinori Nagai[1, 2, *], Hiroe Honda[1, 3], Naoki Okamoto[1], Seiji Yamamoto[4], Takeru Hamashima[4], Yoko Ishii[4], Miyako Tanaka[5], Takayoshi Suganami[2, 5], Masakiyo Sasahara[4], Kensuke Miyake[6, 7], and Kiyoshi Takatsu[1, 3, *]

[1]Department of Immunobiology and Pharmacological Genetics, Graduate School of Medicine and Pharmaceutical Science for Research, University of Toyama, 2630 Sugitani, Toyama-shi, Toyama 930-0194, JAPAN
[2]JST, PRESTO, 4-1-8 Honcho, Kawaguchi, Saitama 332-0012, JAPAN
[3]Toyama Prefectural Institute for Pharmaceutical Research, 17-1 Nakataikouyama, Imizu City, Toyama 939-0363, JAPAN
[4]Department of Pathology, Graduate School of Medicine and Pharmaceutical Science for Research, University of Toyama, 2630 Sugitani, Toyama-shi, Toyama 930-0194, JAPAN
[5]Department of Molecular Medicine and Metabolism, Research Institute of Environmental Medicine, Nagoya University, Furo-cho, Chikusa-ku, Nagoya 464-8601, JAPAN
[6]Divivion of Infectious Genetics, Department of Microbiology and Immunology, [7]Laboratory of Innate Immunity, Center for Experimental Medicine and Systems Biology, The Institute of Medical Science, The University of Tokyo, 4-6-1 Shirokanedai, Minato-ku, Tokyo 108-8639, JAPAN

*Correspondence: Yoshinori Nagai, M.D., Ph.D. and Kiyoshi Takatsu, Ph.D.
Department of Immunobiology and Pharmacological Genetics, Graduate School of Medicine and Pharmaceutical Science for Research, University of Toyama
2630 Sugitani, Toyama-shi, Toyama 930-0194, JAPAN
Phone: +81-76-434-7673
Fax: +81-76-434-5009
E-mail: ynagai@med.u-toyama.ac.jp (Y.N.), takatsuk@med.u-toyama.ac.jp (K.T.)

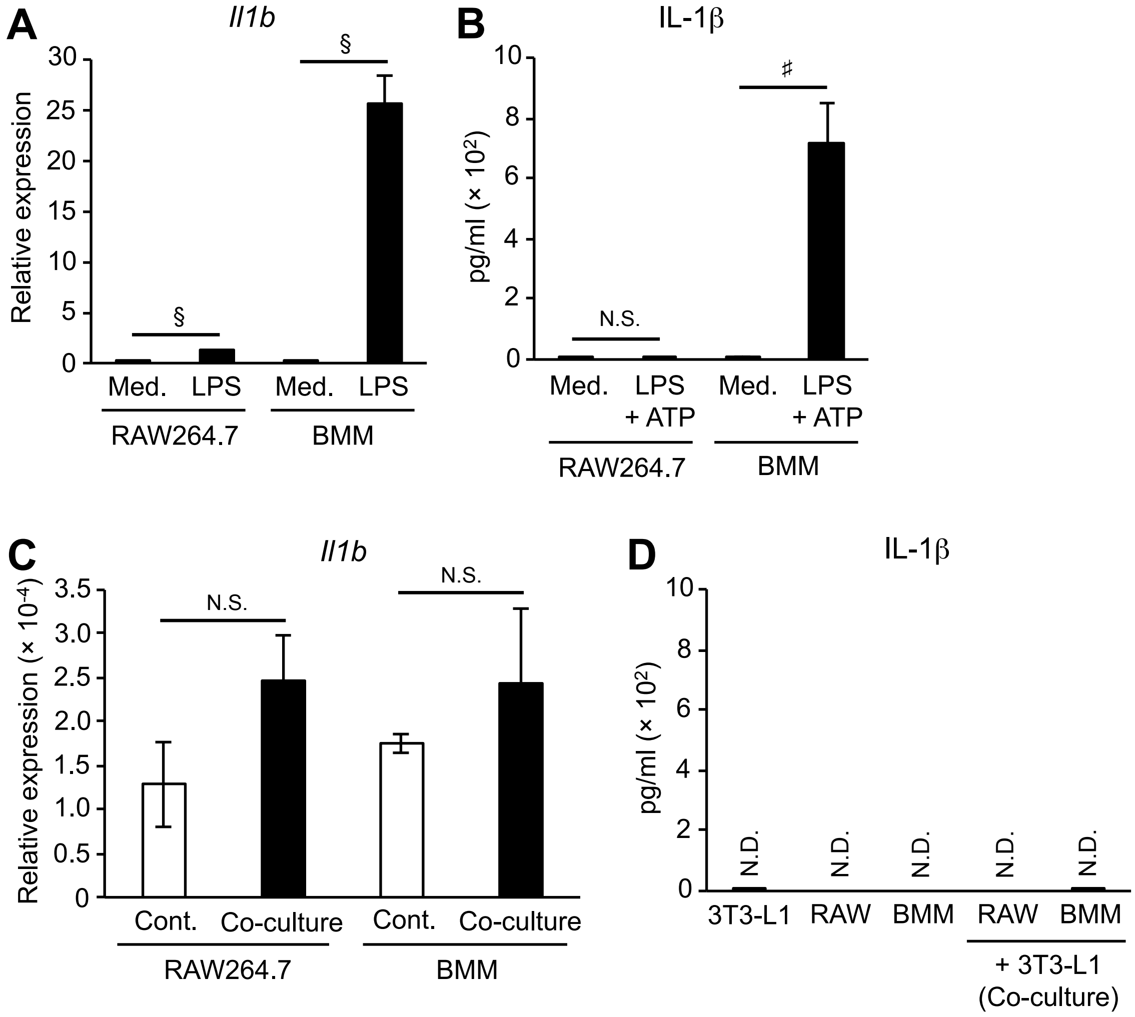

Supplementary Figure 1. The NLRP3 inflammasome activation is not involved in the co-culture of adipocytes and macrophages.

(A) RAW264.7 or BMMs were stimulated with LPS (1 μg/ml) for 3 h (n = 3 per group). IL-1β mRNA expression was determined by RT-qPCR. Data are shown as means ± SD. $^{§}P$ <0.001. (B) RAW264.7 or BMMs were stimulated with LPS (1 μg/ml) for 3 h, followed by the stimulation of ATP (2 mM) for 40 min (n = 3 per group). IL-1β production in the culture supernatants was measured by ELISA. N.S., not significant. $^{\#}P$ <0.01. (C) Differentiated 3T3-L1 adipocytes were co-cultured with RAW264.7 macrophages or BMMs for 24 h (n = 3 per group). IL-1β mRNA expression was determined by RT-qPCR. Data are shown as means ± SD. N.S., not significant. (D) RAW264.7 or BMMs were co-cultured with differentiated 3T3-L1 adipocytes for 24 h (n = 3 per group). As control cultures, RAW264.7, BMMs, or differentiated 3T3-L1 adipocytes alone was cultured for 24 h. IL-1β production in the culture supernatants was measured by ELISA. N.D., not detected.

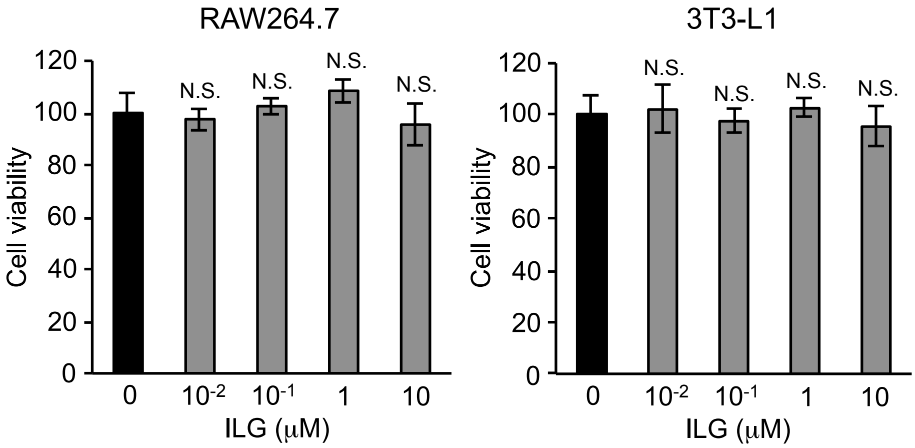

Supplementary Figure 2. ILG has no effect on cell viability of RAW264.7 macrophages and differentiated 3T3-L1 adipocytes.

RAW264.7 or differentiated 3T3-L1 adipocytes were left untreated or treated with the indicated concentrations of ILG for 24 h. Cell viability assay was conducted by using a Cell Titer 96® Aqueous One Solution Cell Proliferation Assay as described in Methods. Data are shown as means ± SD of triplicate wells and representative of three independent experiments. N.S., not significant.

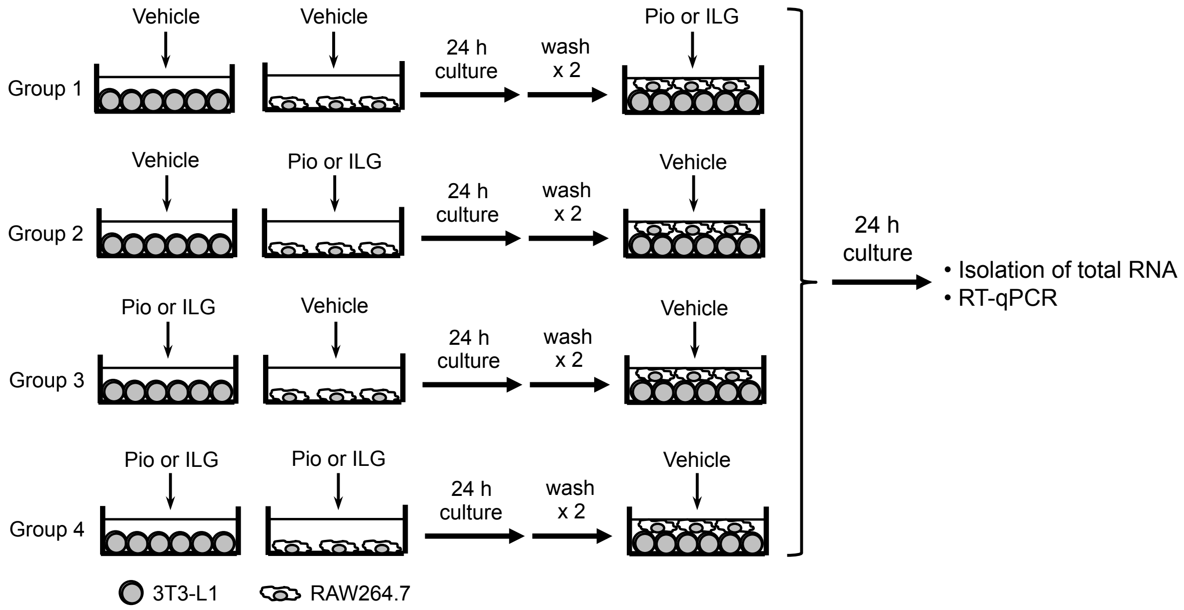

Supplementary Figure 3. Schematic diagram of the co-culture of differentiated 3T3-L1 adipocytes and RAW264.7 macrophages.

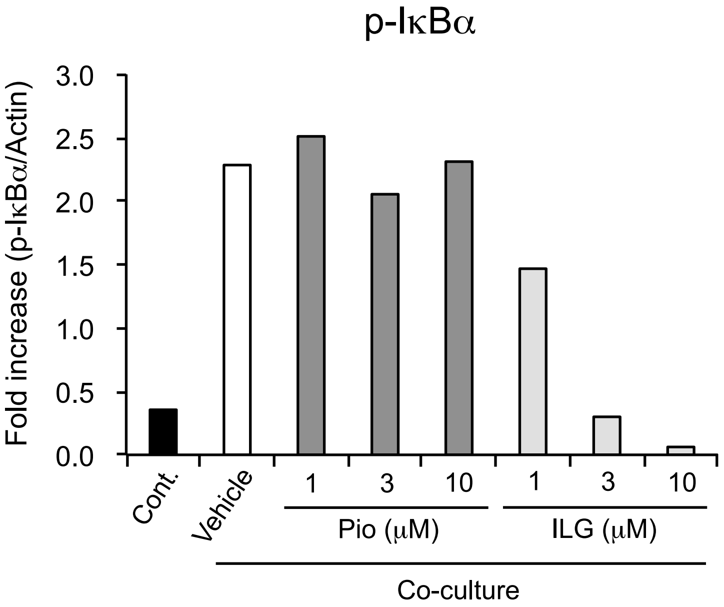

Supplementary Figure 4. Quantitative analysis of phosphorylated IκBα in each group of Figure 2C.

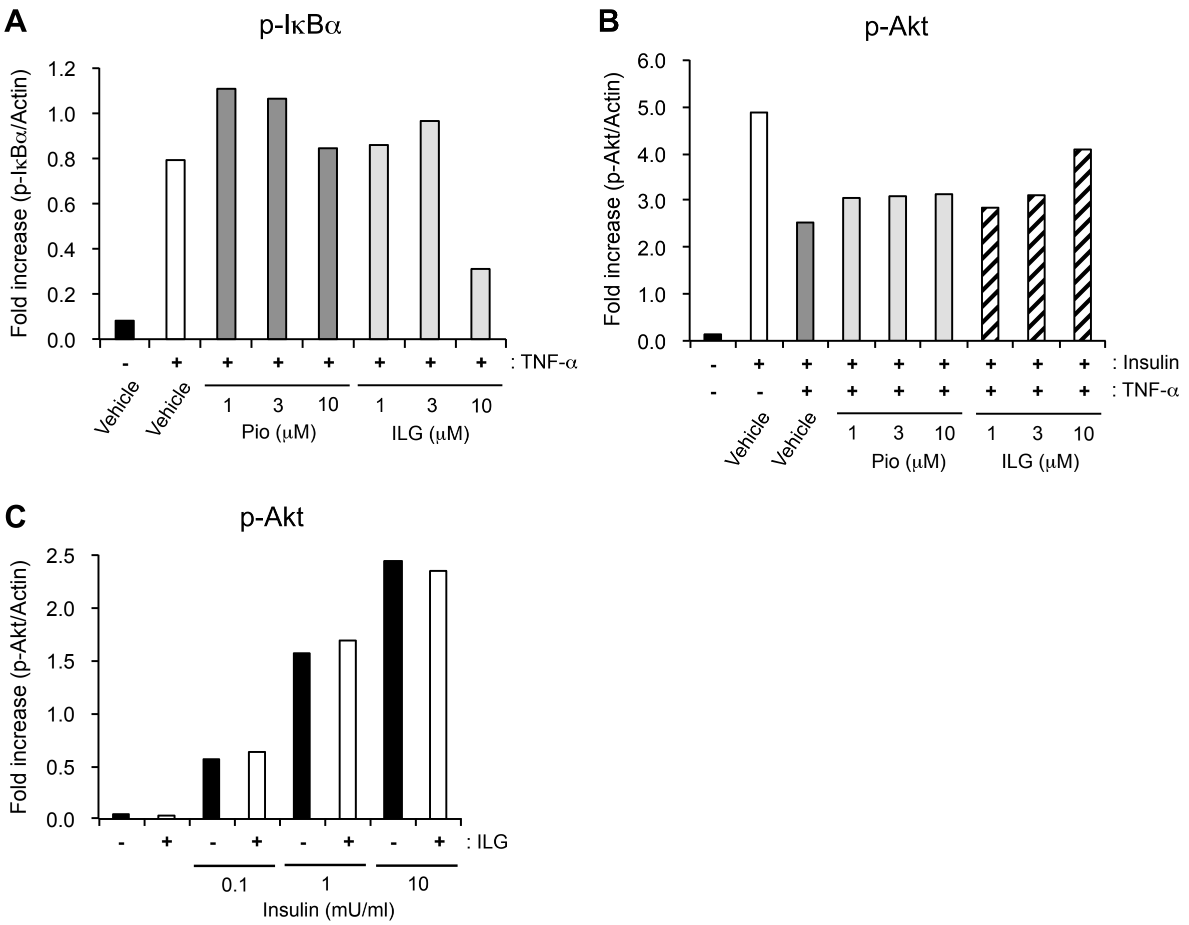

Supplementary Figure 5. Quantitative analyses of phosphorylated IκBα (A) and Akt (B, C) in each group of Figure 3C, 3D, and 3E.

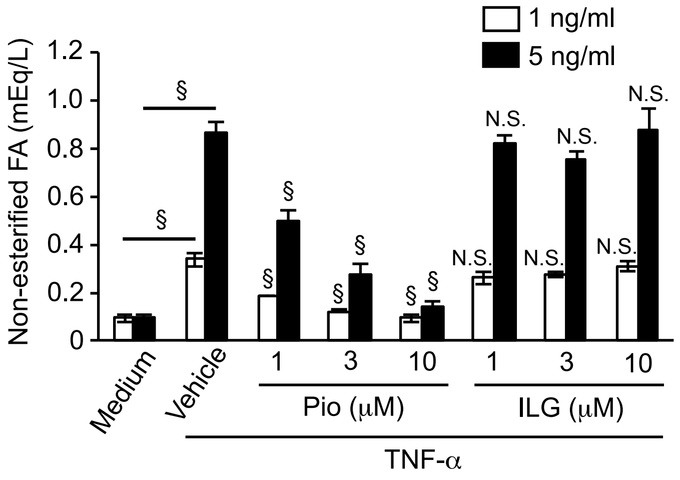

Supplementary Figure 6. ILG has no effect on TNF-α-induced FA release in differentiated 3T3-L1 adipocytes.

Differentiated 3T3-L1 adipocytes were treated with pioglitazone (Pio) or ILG for 6 h and subsequently stimulated with TNF-α (1 or 5 ng/ml) for 24 h (n = 3 per group). Non-esterified FA in the culture supernatants was measured as described in Methods. Data are shown as means ± SD. N.S., not significant. $^{\S}P$ <0.001.

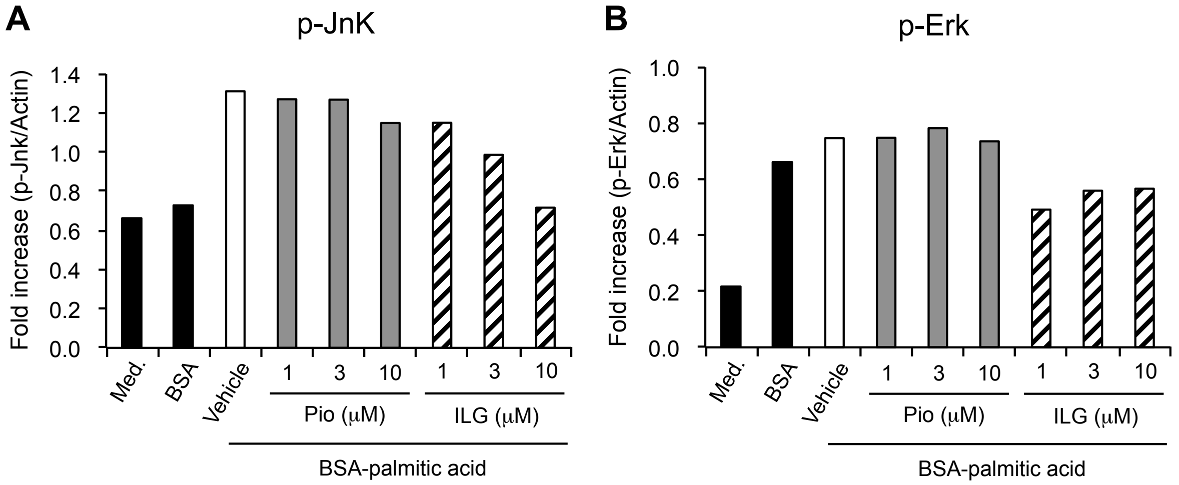

Supplementary Figure 7. Quantitative analyses of phosphorylated Jnk (A) and Erk (B) in each group of Figure 4D.

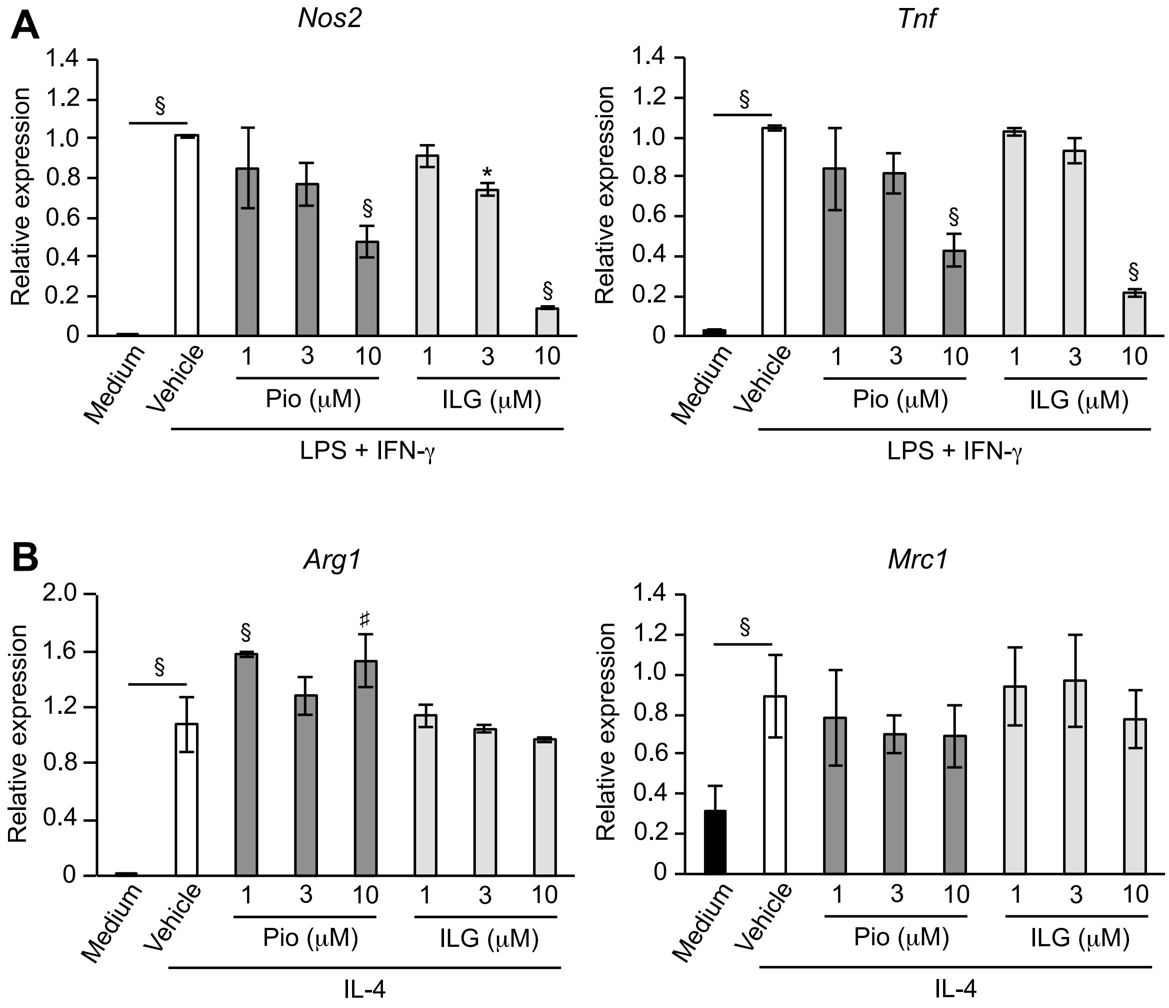

Supplementary Figure 8. ILG attenuates M1 but not M2 macrophage differentiation *in vitro*.

(A) BMMs were left untreated or treated with pioglitazone (Pio) or ILG for 30 min and subsequently stimulated with LPS (1 ng/ml) plus IFN-γ (20 ng/ml) for 24 h (n = 3 per group). iNOS and TNF-α mRNA expression were measured by RT-qPCR. Data are shown as means ± SD. $^{*}P$ <0.05, $^{\S}P$ <0.001. (B) BMMs were left untreated or treated with pioglitazone (Pio) or ILG for 30 min and subsequently stimulated with IL-4 (20 ng/ml) for 24 h (n = 3 per group). Arginase-1 and CD206 mRNA expression were measured by RT-qPCR. Data are shown as means ± SD. $^{\#}P$ <0.01, $^{\S}P$ <0.001. All data are representative of at least three independent experiments.

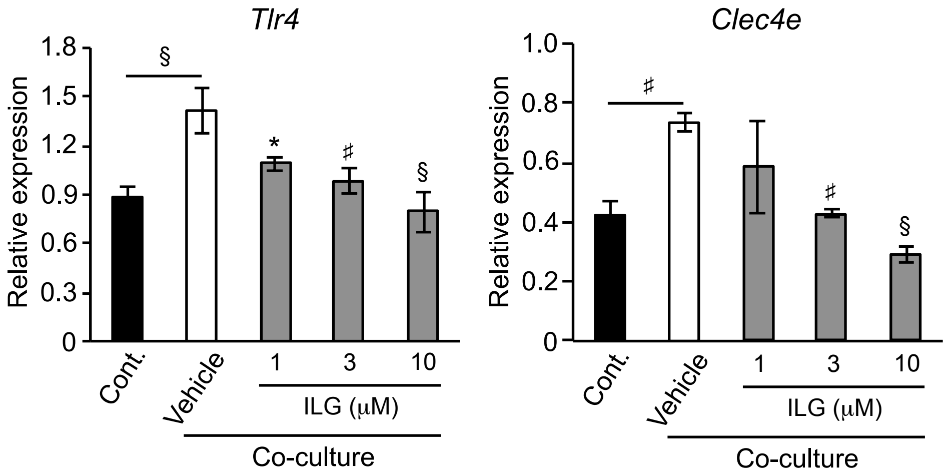

Supplementary Figure 9. ILG decreases expression of TLR4 and Mincle mRNA in the co-cultured cells.

Differentiated 3T3-L1 adipocytes were co-cultured with RAW264.7 macrophages in the presence of ILG or vehicle for 24 h (n = 3 per group). TLR4 and Mincle mRNA expression were determined by RT-qPCR. Data are shown as means ± SD. $^{*}P < 0.05$, $^{\#}P < 0.01$, $^{\S}P < 0.001$. Data are representative of at least two independent experiments.

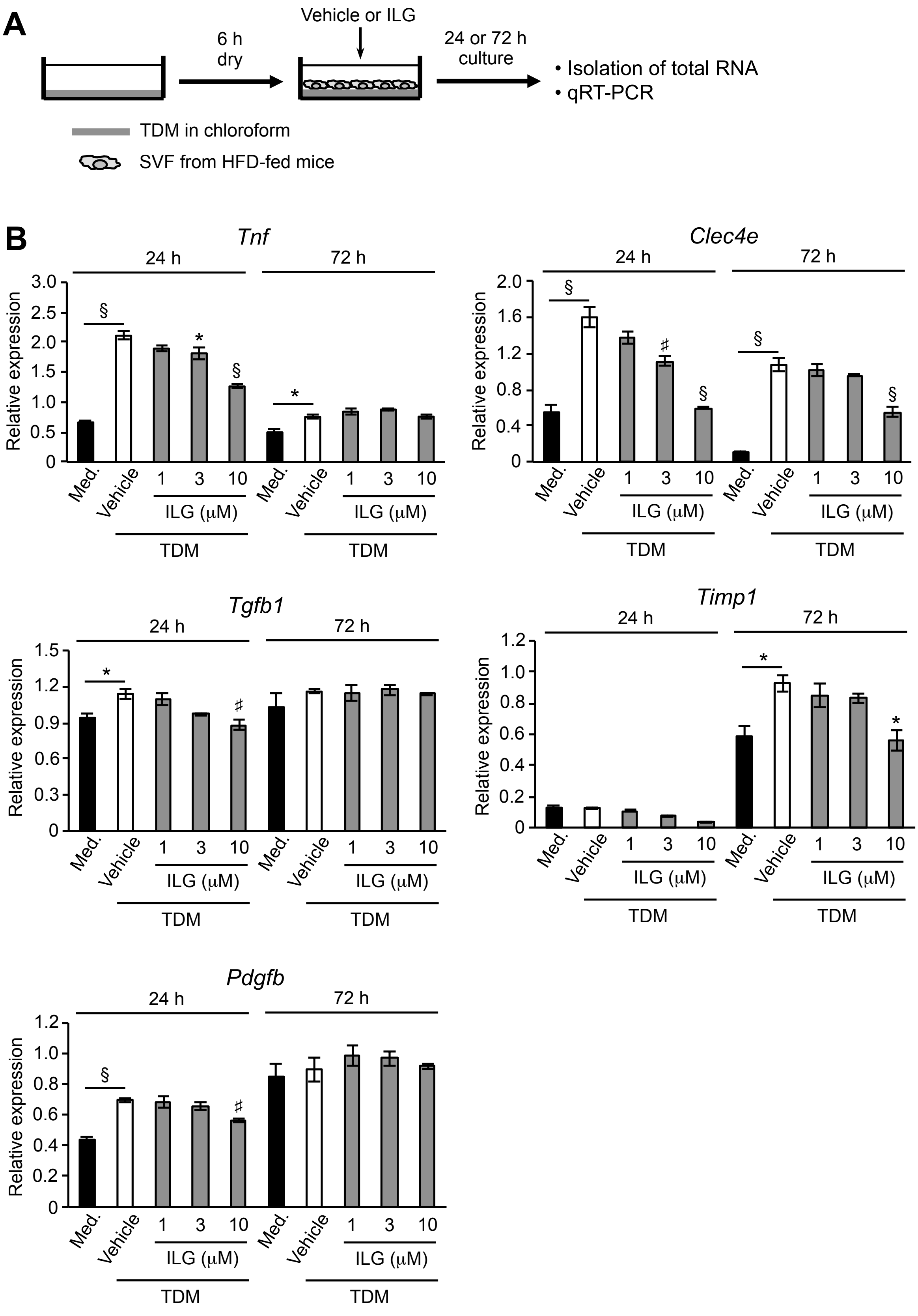

A
Vehicle or ILG
6 h
dry
24 or 72 h
culture
• Isolation of total RNA
• qRT-PCR
TDM in chloroform
SVF from HFD-fed mice
B
Tnf
Clec4e
Tgfb1
Timp1
Pdgfb
24 h
72 h
Relative expression
Med.
Vehicle
ILG (μM)
TDM

Supplementary Figure 10. ILG attenuates Mincle-stimulated expression of fibrosis-related genes in SVF.

(A) Schematic diagram of the stimulation of SVF with TDM. SVF from C57BL/6 mice fed with an HFD for 12 weeks was stimulated with TDM (2.5 μg/well) in the presence of vehicle or ILG for 24 or 72 h. (B) RT-qPCR for TNF-α, Mincle, TGF-β, TIMP-1, and PDGF-B mRNA in the TDM-stimulated SVF with or without ILG (n = 3 per group). Data are shown as means ± SE. $^{*}P$ <0.05, $^{\#}P$ <0.01, $^{\S}P$ <0.001. Data are representative of at least two independent experiments.

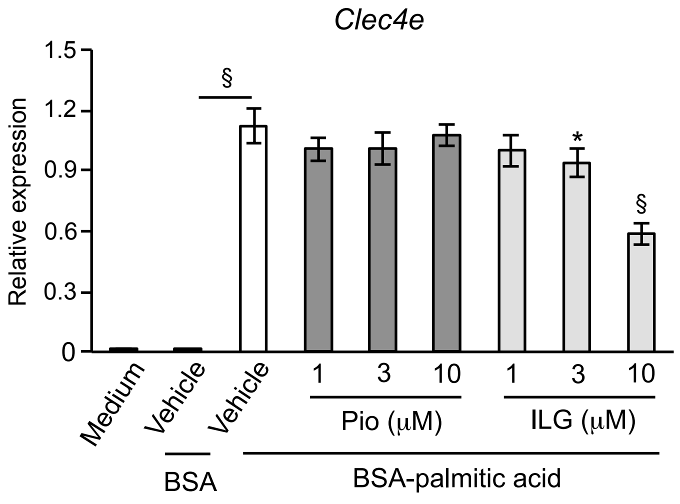

Supplementary Figure 11. ILG attenuates palmitic acid-induced Mincle expression in macrophages.

BMMs were treated with pioglitazone (Pio) or ILG for 6 h and subsequently stimulated with BSA-palmitic acid (200 μM) for 24 h (n = 3 per group). Mincle mRNA expression was measured by RT-qPCR. Data are shown as means ± SD. $^{*}P$ <0.05, $^{§}P$ <0.001. Data are representative of at least three independent experiments.

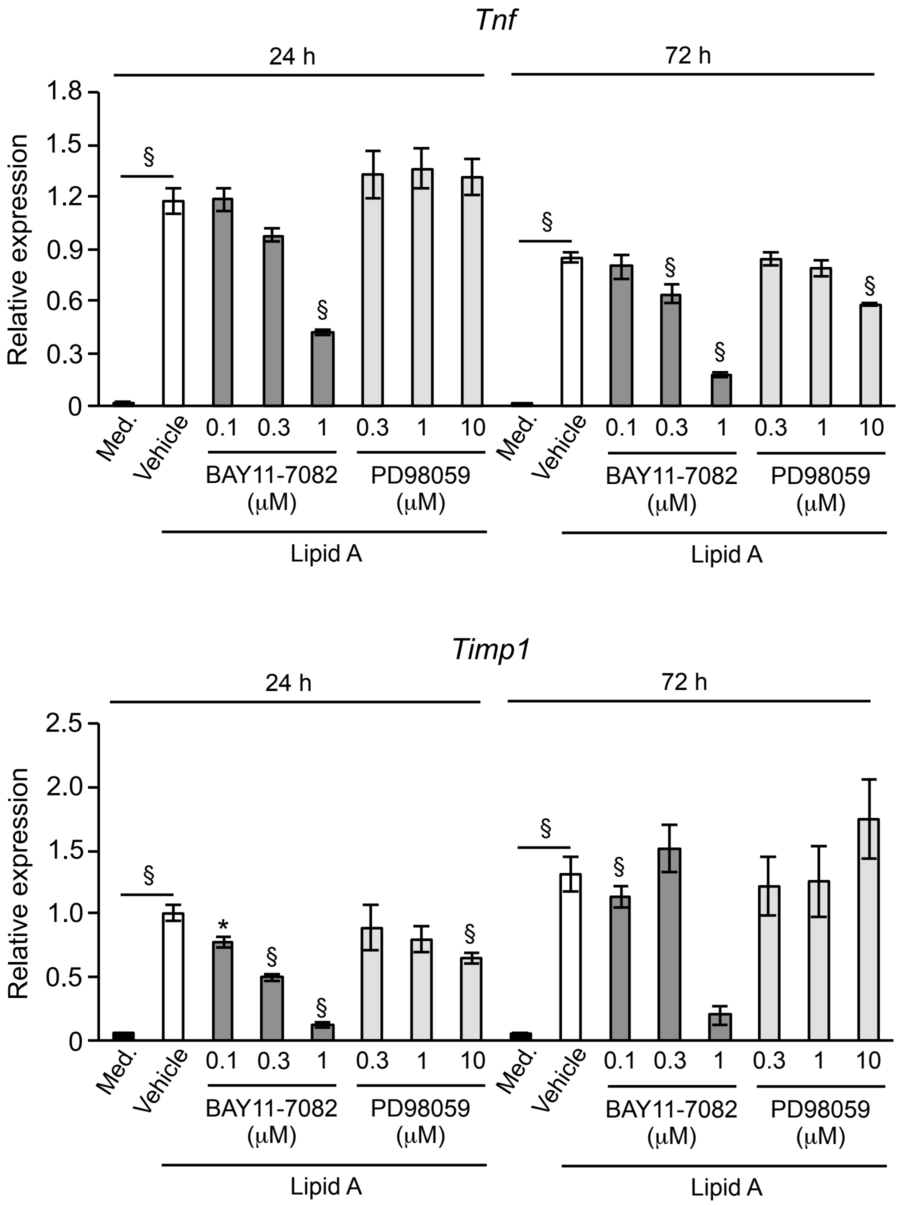

Supplementary Figure 12. A NF-κB inhibitor potently suppresses lipid A-induced TNF-α and TIMP-1 mRNA expression in macrophages.

RT-qPCR for TNF-α and TIMP-1 mRNA in the lipid A-stimulated macrophages with or without indicated concentrations of BAY11-7082 or PD98059 (n = 3 per group). Thioglycolate-elicited peritoneal macrophages were left untreated or treated with BAY11-7082 or PD98059 for 30 min and subsequently stimulated with lipid A (1 μg/ml) for 24 or 72 h. Data are shown as means ± SD. $^{*}P < 0.05$, $^{§}P < 0.001$. Data are representative of at least two independent experiments.

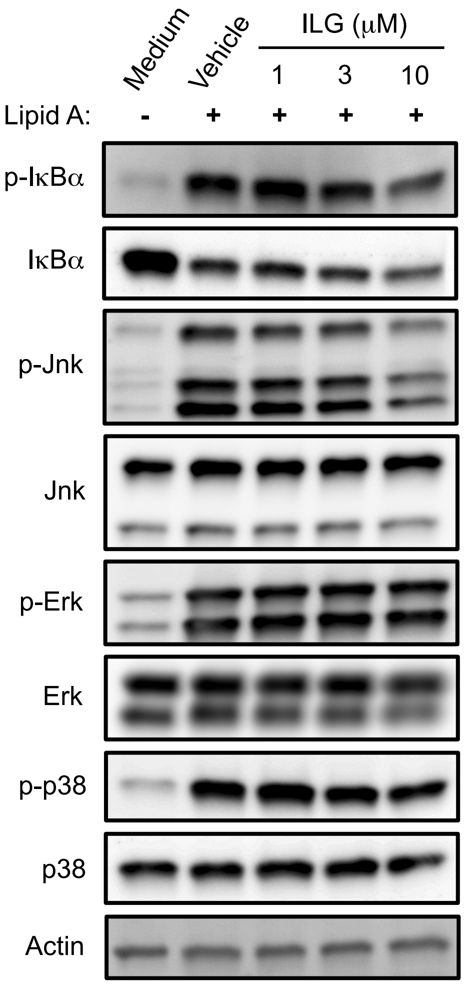

Supplementary Figure 13. ILG attenuates lipid A-induced phosphorylation of IκBα, Jnk, and p38 in macrophages.

Thioglycolate-elicited peritoneal macrophages from C57BL/6 mice were treated with vehicle or ILG for 6 h and subsequently stimulated with lipid A for 30 or 60 h. Phosphorylation (p) of IκBα, Jnk, Erk, and p38 was examined by western blotting. All data are representative of at least two independent experiments.

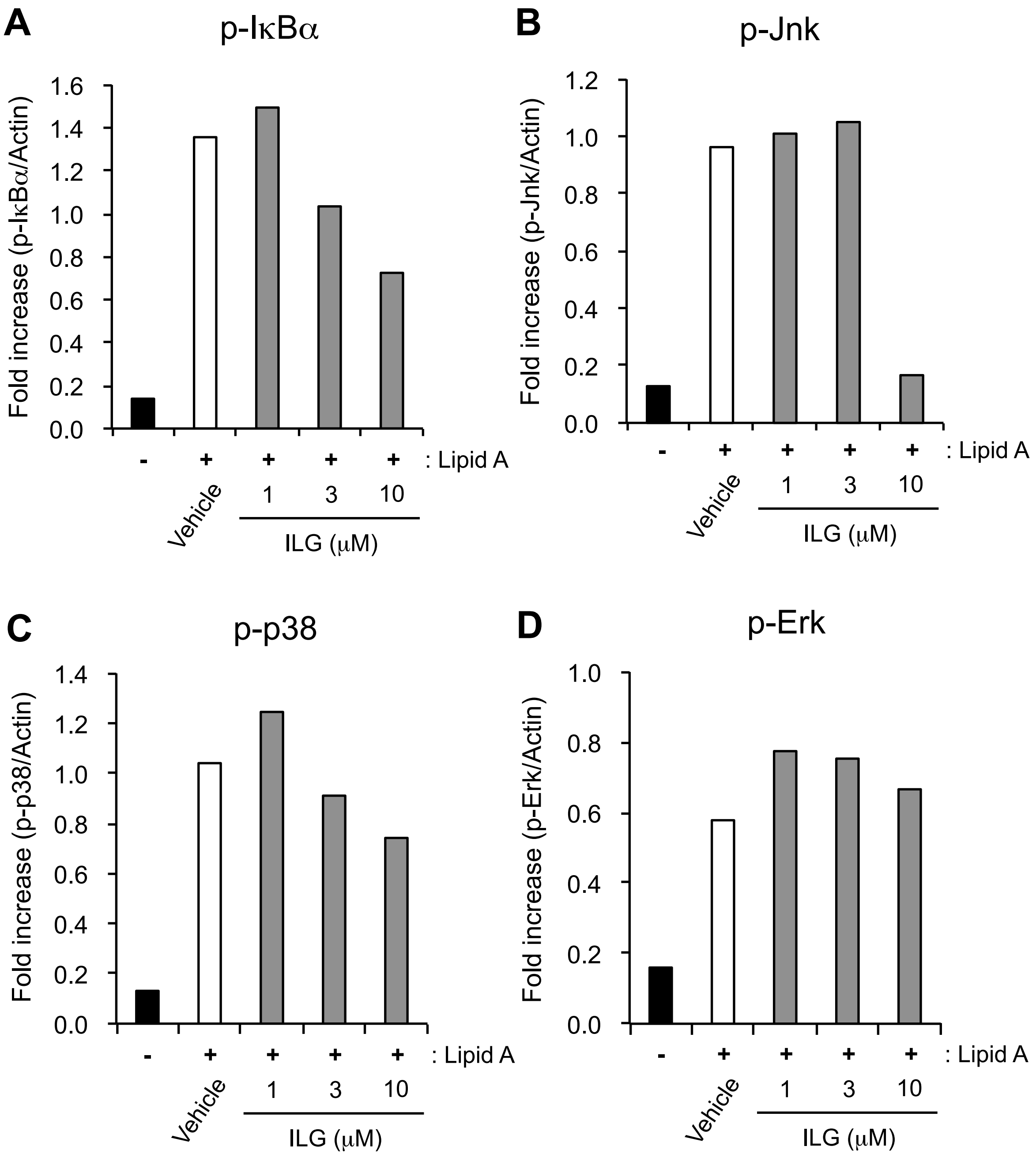

Supplementary Figure 14. Quantitative analyses of phosphorylated IκBα (A), Jnk (B), p38 (C), and Erk (D) in each group of Supplementary Figure 13.

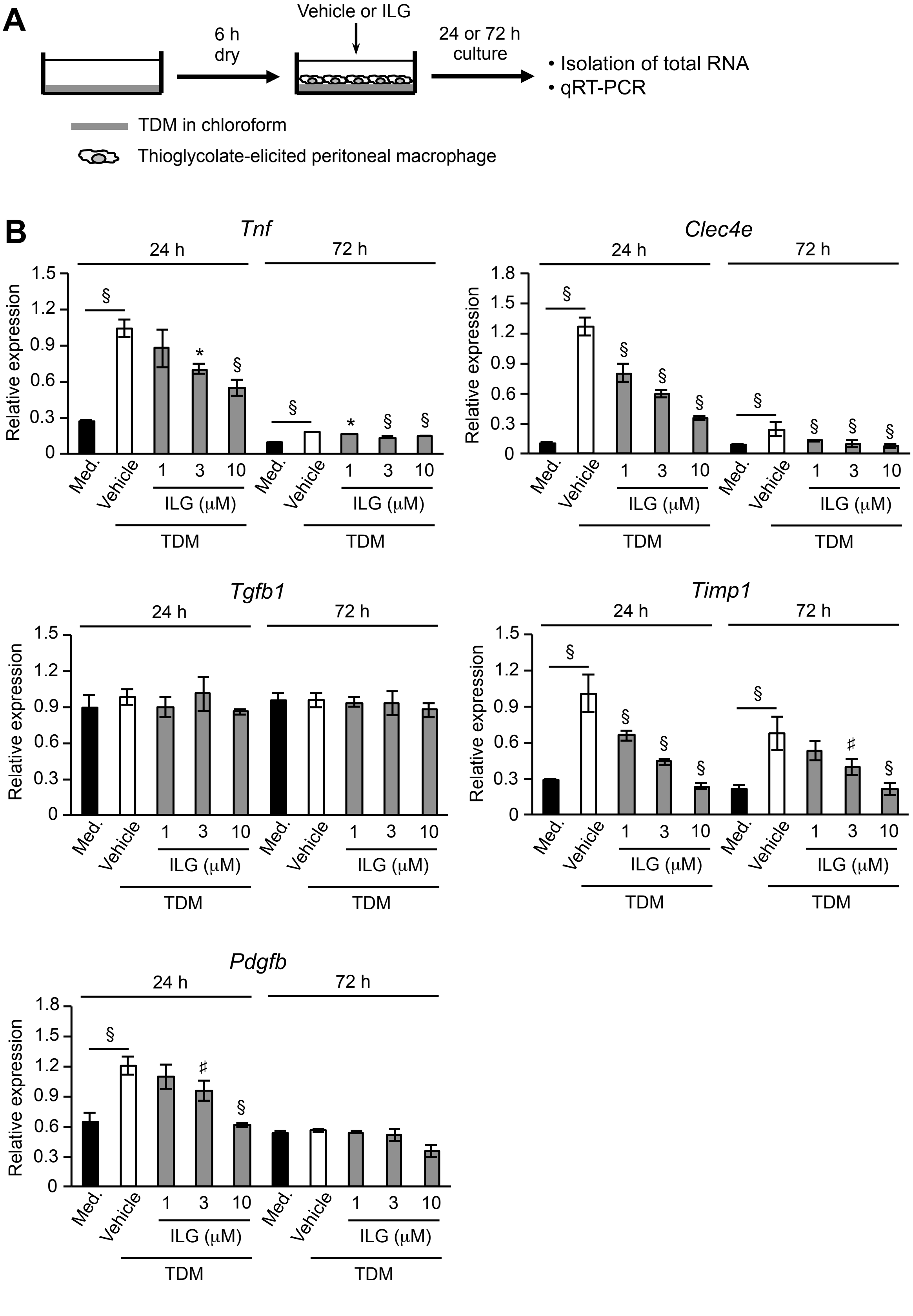
A
Vehicle or ILG
6 h
dry
24 or 72 h
culture
• Isolation of total RNA
• qRT-PCR
TDM in chloroform
Thioglycolate-elicited peritoneal macrophage
B
Tnf
Clec4e
Tgfb1
Timp1
Pdgfb
24 h
72 h
Relative expression
Med.
Vehicle
ILG (μM)
TDM

Supplementary Figure 15. ILG attenuates Mincle-stimulated expression of fibrosis-related genes in macrophages.

(A) Schematic diagram of the stimulation of macrophages with TDM. Thioglycolate-elicited peritoneal macrophages from C57BL/6 mice were stimulated with TDM (2.5 μg/well) in the presence of vehicle or ILG for 24 or 72 h. (B) RT-qPCR for TNF-α, Mincle, TGF-β, TIMP-1, and PDGF-B mRNA in the TDM-stimulated macrophages with or without ILG (n = 3 per group). Data are shown as means ± SE. $^{*}P$ <0.05, $^{\#}P$ <0.01, $^{\S}P$ <0.001. Data are representative of at least two independent experiments.

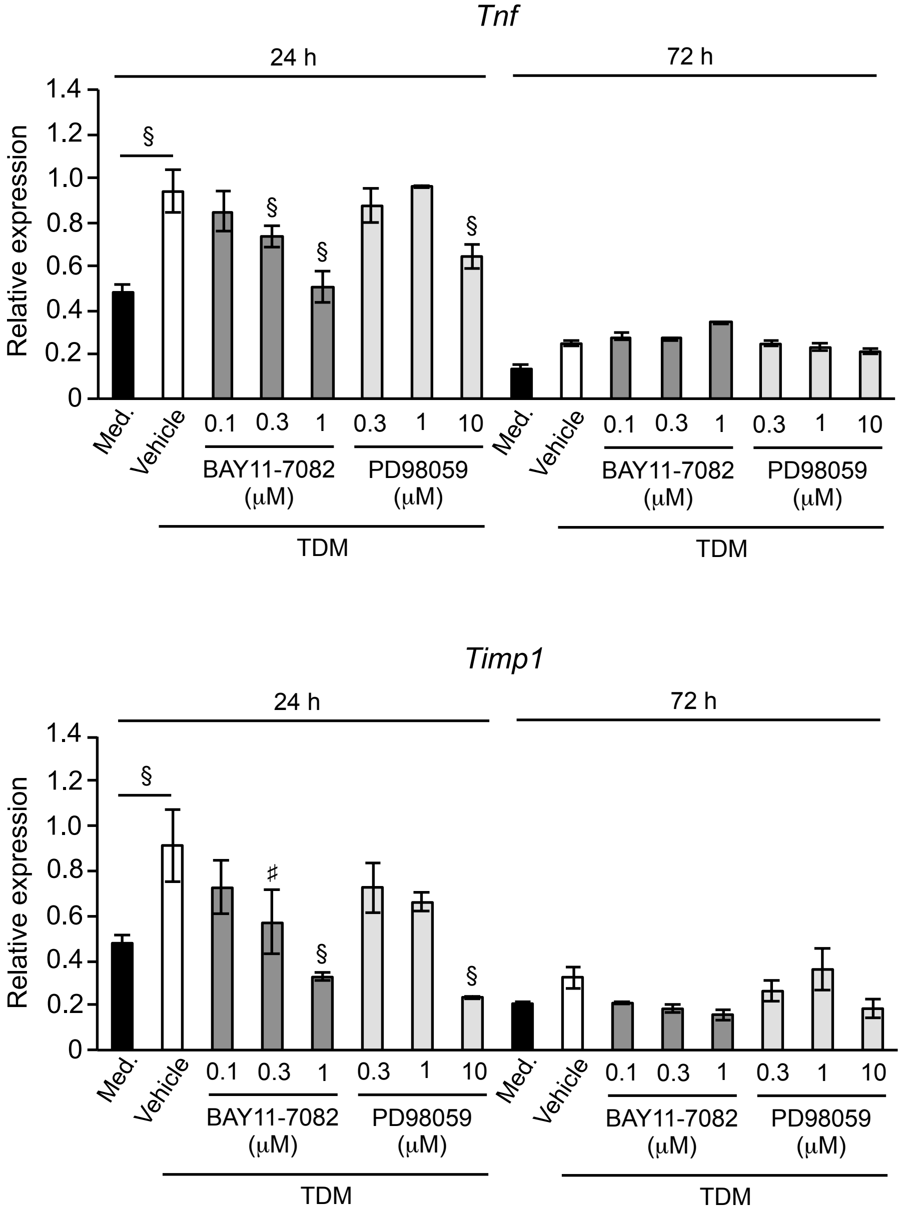

Supplementary Figure 16. A NF-κB inhibitor potently suppresses TDM-induced TNF-α and TIMP-1 mRNA expression in macrophages.

RT-qPCR for TNF-α and TIMP-1 mRNA in the TDM-stimulated macrophages with or without BAY11-7082 or PD98059 (n = 3 per group). Thioglycolate-elicited peritoneal macrophages were stimulated with TDM (2.5 μg/well) for 24 or 72 h in the presence of indicated concentrations of BAY11-7082 or PD98059. Data are shown as means ± SD. $^{\#}P$ <0.01, $^{\S}P$ <0.001. Data are representative of at least two independent experiments.

Gene	Gene Symbol	Gene Name	Assay ID
Hprt	Hprt1	hypoxanthine guanine phosphoribosyl transferase	Mm00446968_m1
TNF-a	Tnf	tumor necrosis factor	Mm00443258_m1
MCP-1	Ccl2	chemokine (C-C motif) ligand 2	Mm00441243_g1
Il-1b	Il1b	interleukin 1 beta	Mm01336189_m1
iNOS	Nos2	nitric oxide synthase 2, inducible	Mm01309898_m1
Arg-1	Arg1	arginase, liver	Mm01190441_g1
CD206	Mrc1	mannose receptor, C type 1	Mm00485148_m1
Tgfb-1	Tgfb1	transforming growth factor, beta 1	Mm01178820_m1
TIMP-1	Timp1	tissue inhibitor of metalloproteinase 1	Mm00441818_m1
Tlr4	Tlr4	toll-like receptor 4	Mm00445273_m1
PDGF-B	Pdgfb	platelet derived growth factor, B polypeptide	Mm00440677_m1
Col1a-1	Col1a1	collagen, type 1, alpha 1	Mm00801666_g1
Mincle	Clec4e	C-type lectin domain family 4, member e	Mm01183703_m1

Supplementary Table 1. RT-qPCR primers. The primers were purchased from Applied Biosystems.
